# PGL I expression in live bacteria allows activation of a CD206/PPARγ cross-talk that may contribute to successful *Mycobacterium leprae* colonization of peripheral nerves

**DOI:** 10.1371/journal.ppat.1007151

**Published:** 2018-07-06

**Authors:** Chyntia Carolina Díaz Acosta, André Alves Dias, Thabatta Leal Silveira Andrezo Rosa, Leonardo Ribeiro Batista-Silva, Patricia Sammarco Rosa, Thiago Gomes Toledo-Pinto, Fabrício da Mota Ramalho Costa, Flávio Alves Lara, Luciana Silva Rodrigues, Katherine Antunes Mattos, Euzenir Nunes Sarno, Patrícia Torres Bozza, Christophe Guilhot, Márcia de Berrêdo-Pinho, Maria Cristina Vidal Pessolani

**Affiliations:** 1 Laboratory of Cellular Microbiology, Oswaldo Cruz Institute, Rio de Janeiro, RJ, Brazil; 2 Lauro de Souza Lima Institute, Bauru, SP, Brazil; 3 Leprosy Laboratory, Oswaldo Cruz Institute, Rio de Janeiro, RJ, Brazil; 4 Laboratory of Immunopharmacology, Oswaldo Cruz Institute, Rio de Janeiro, RJ, Brazil; 5 Institut de Pharmacologie et de Biologie Structurale, IPBS, Université de Toulouse, CNRS, UPS, Toulouse, France; McGill University Health Centre, CANADA

## Abstract

*Mycobacterium leprae*, an obligate intracellular bacillus, infects Schwann cells (SCs), leading to peripheral nerve damage, the most severe leprosy symptom. In the present study, we revisited the involvement of phenolic glycolipid I (PGL I), an abundant, private, surface *M*. *leprae* molecule, in *M*. *leprae*-SC interaction by using a recombinant strain of *M*. *bovis* BCG engineered to express this glycolipid. We demonstrate that PGL I is essential for bacterial adhesion and SC internalization. We also show that live mycobacterium-producing PGL I induces the expression of the endocytic mannose receptor (MR/CD206) in infected cells in a peroxisome proliferator-activated receptor gamma (PPARγ)-dependent manner. Of note, blocking mannose recognition decreased bacterial entry and survival, pointing to a role for this alternative recognition pathway in bacterial pathogenesis in the nerve. Moreover, an active crosstalk between CD206 and the nuclear receptor PPARγ was detected that led to the induction of lipid droplets (LDs) formation and prostaglandin E2 (PGE2), previously described as fundamental players in bacterial pathogenesis. Finally, this pathway was shown to induce IL-8 secretion. Altogether, our study provides evidence that the entry of live *M*. *leprae* through PGL I recognition modulates the SC phenotype, favoring intracellular bacterial persistence with the concomitant secretion of inflammatory mediators that may ultimately be involved in neuroinflammation.

## Author summary

Nerve damage is the most severe symptom of leprosy, an ancient disease that continues to be a major health problem in several countries. Nerve damage is due to the ability of *Mycobacterium leprae*, the etiologic agent, to invade SCs, the glial cells of the peripheral nervous system. Understanding the molecular basis of *M*. *leprae–*SC interaction is essential for the creation of new tools aiming to treat and, above all, prevent leprosy neuropathy. This study demonstrates the critical role of PGL I, an *M*. *leprae*-abundant specific cell wall lipid, in establishing infection. PGL I is not only a prerequisite in initiating bacterial adhesion to and subsequent invasion of SCs, but also for changing the repertoire of cell surface proteins to allow for the entrance of bacteria via alternative pathways. These new invasive pathways induced by PGL I involve recognition of other bacterial cell surface glycolipids that, in turn, evoke functional changes in the infected cell, including the accumulation of host cell-derived lipids, which favor bacterial survival. These pathways also promote the secretion of inflammatory mediators that may contribute to nerve damage. In an era of translational-oriented research, exploring these receptors in depth could lead to the development of attractive strategies to ensure the targeted intracellular delivery of therapeutics aiming to prevent neuropathy.

## Introduction

The most serious consequence of leprosy is the peripheral nerve damage that occurs in all clinical forms of the disease. Nerve damage results from the capacity of *M*. *leprae*, an obligate intracellular bacillus, to infect SCs, the glial cells of the peripheral nervous system (PNS). SCs show remarkable plasticity and contribute to the regenerative capacity of the adult PNS even after severe injury has occurred. *M*. *leprae*-nerve fiber colonization results in loss of sensation, an early symptom of the disease. While multidrug therapy (MDT) treats the infection, it may be unsuccessful in either preventing or arresting the nerve damage responsible for disfigurement and disabilities [1, 2]. In-depth investigation of *M*. *leprae*-nerve interactions objectifying the development of new strategies for the prevention and treatment of leprosy-related nerve impairments is, therefore, of utmost importance. *M*. *leprae* is easily seen inside vacuoles in the non-myelinating and myelinating SC cytoplasm in nerve specimens of leprosy patients [3,4] and, as a consequence, the three physiological functions of nerves–sensory, motor and autonomic–are affected. However, the first symptoms of leprosy are related to loss of temperature sensation and decreased touch sensation, functions provided by non-myelinating fibers, indicating their early invasion by the leprosy bacillus during the natural course of the disease [5]. Thus, the use of non-myelinating SCs as an in vitro model of infection is physiologically relevant and may reveal early fundamental aspects of *M*. *leprae* neuropathogenesis.

**Fig 1:**
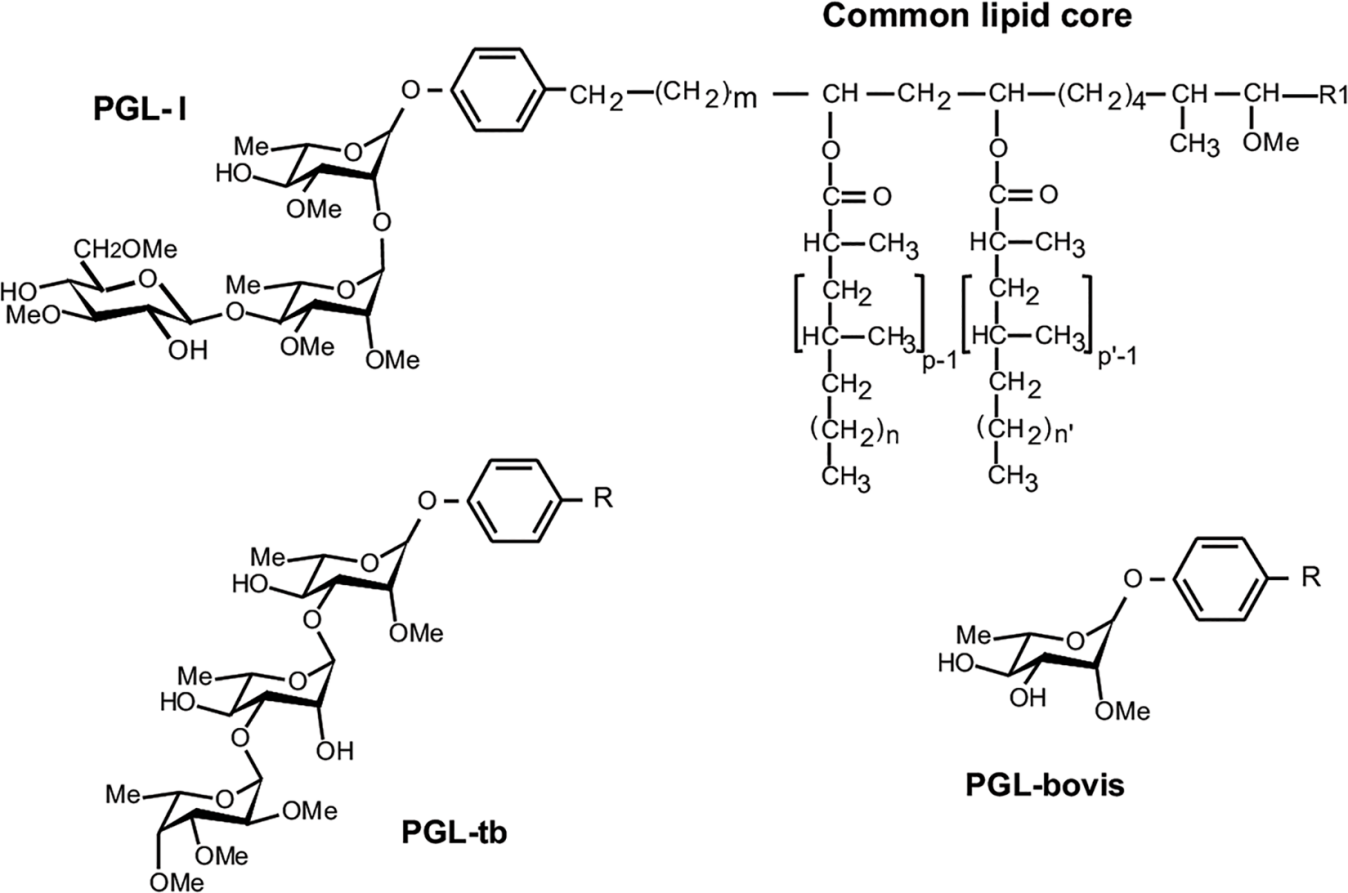
Molecular structures of mycobacterium phenolic glycolipids. The structures of the phenolic glycolipids from *M*. *leprae* (PGL I), *M*. *tuberculosis* (PGL-tb), and *M*. *bovis* (PGL-bovis) highlight the differences in the saccharidic moiety. R = common lipid core. In [14–16].

The tropism of *M*. *leprae* to the peripheral nerves has been attributed to its capacity to bind to the globular domain of the α2 chain of laminin-2 [6], a laminin isotype with restricted tissue distribution constituting a major component of the basal lamina surrounding SC-axon units [7–9]. Two *M*. *leprae* adhesins named Hlp and PGL I have been characterized as laminin-binding molecules responsible for attachment to SC. Shimoji *et al*.[10] and Marques *et al*.[11] have described the 21-kDa histone-like protein (Hlp), a conserved molecule among species of mycobacteria, as a laminin-binding protein (also called ML-LBP21). The other molecule, PGL I [12] is an abundant lipid composing the outermost layer of the *M*. *leprae* envelope [13]. PGLs are present in other species of mycobacteria, but differ among themselves in their carbohydrate moieties [14–16] (Fig 1). The PGL I trisaccharide is highly specific to *M*. *leprae* [13, 17], having been shown to bind exclusively to the G domain of the laminin-2 α2 chain [12]. It has been proposed that this interaction most convincingly explains the specific neural tropism displayed by *M*. *leprae* since Hlp can also bind the α1 and β1 chains that make up other laminin isotypes [10,11,18,19] such as laminin-1 with a wide range of tissue distribution [7]. It has been speculated that, due to its abundance, PGL I would then initiate the specific *M*. *leprae*-SC interaction while Hlp would increase avidity binding by enacting a secondary role [12].

Upon infection, *M*. *leprae* seems to evoke drastic metabolic and phenotypic changes in SC, abetting infection and bacterial persistence in the host. In previous studies, we were able to show that *M*. *leprae* induces the production of insulin-like growth factor 1 (IGF-1) to advantage host cell survival [20] while triggering drastic changes in the SC lipid and glucose metabolism that promote bacterial persistence [21–23]. Moreover, recent data have shown the capacity of *M*. *leprae* to induce SC reprogramming to a progenitor/stem-like cell stage, most probably increasing the spread of infection [24]. However, the molecular mechanisms underlying these events and the contributions of such *M*. *leprae* constituents as PGL I continue to be poorly understood.

Multiple attempts to grow *M*. *leprae* in axenic or tissue cultures have been in vain so that infected nine-banded armadillo and athymic nude mouse still constitute the major bacterial sources for both biochemical and functional studies [2]. Since mutated strains of *M*. *leprae* are yet unobtainable, a genetically-engineered *M*. *bovis* BCG strain producing and secreting PGL I (BCG PGL I) instead of its own PGL was created as an alternative tool to better decipher the role of this glycolipid in leprosy pathogenesis [15]. Tabouret *et al*. [15] used the recombinant BCG strain to analyze the role of PGL I in the interaction of the leprosy bacillus with macrophages and dendritic cells. As a result, previous data were confirmed and new insights into the molecular bases of PGL I to down modulate the innate immune response were provided. Here, the recombinant BCG PGL I was used as an alternative tool to elucidate the role of PGL I along with the molecular mechanisms by which it participates in the *M*. *leprae*-SC interaction.

## Results

### PGL I production and bacterial viability are determinants for mycobacterial internalization into human Schwann cells

**Fig 2:**
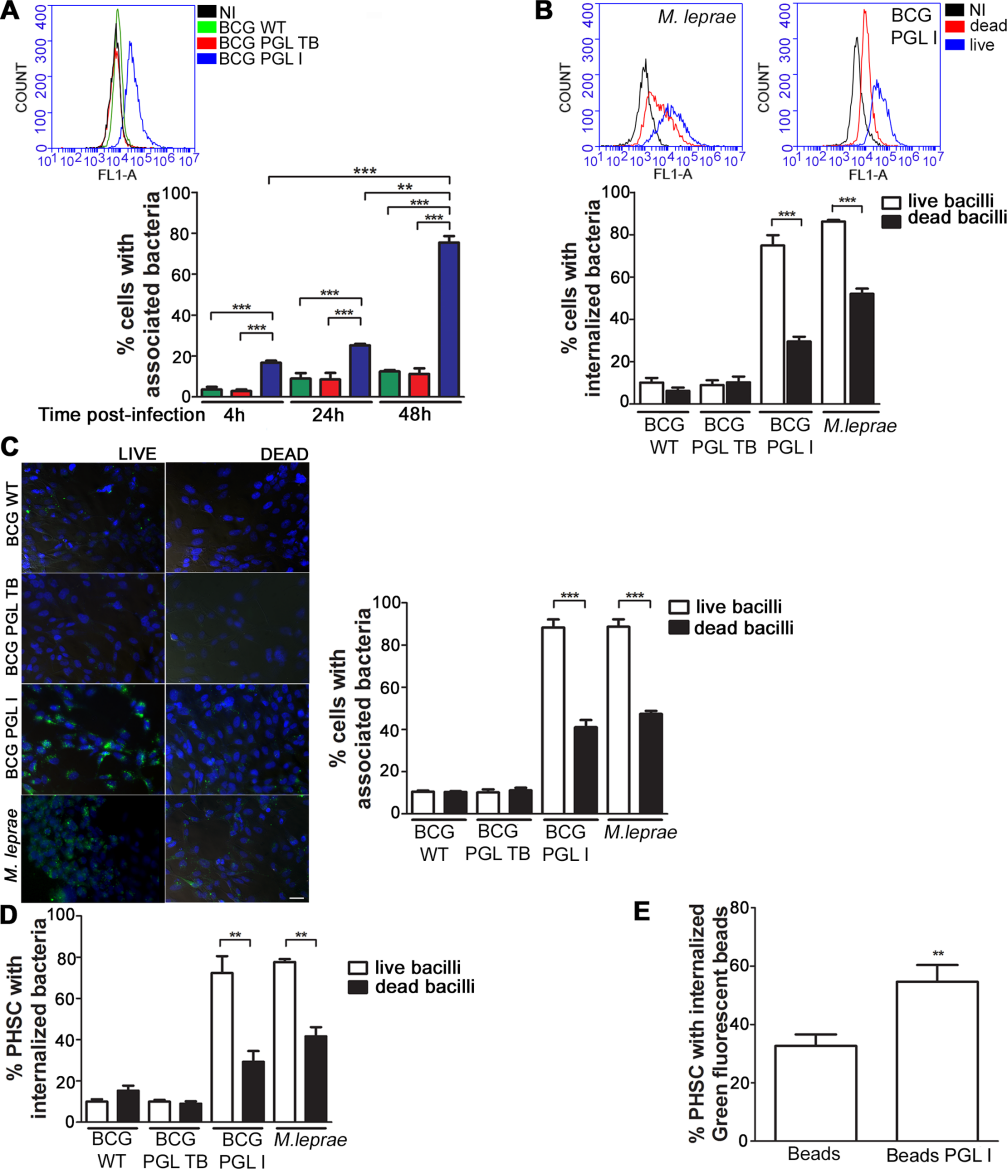
PGL I production and bacterial viability are determinants for mycobacterial internalization into Schwann cells. **A.** The level of bacterial association of the PKH67-(green) labeled BCG recombinant strains was determined by Flow Cytometry (FL1-A green channel). ST8814 SC were either left uninfected (NI) or were treated with BCG WT, BCG PGL TB, or BCG PGL I. A representative histogram plot of the 48 h incubation experiment is shown. The percentage of bacterial association was determined after 4 h, 24 h, and 48 h of incubation with SC at 33°C, MOI 50:1. **B.** Association and internalization of live and dead bacilli were determined by flow cytometry after 48 h of incubation at 33°C and MOI 50:1. Bacteria were labeled with PKH67 and the degree of internalization was determined after Trypan Blue quenching. Representative histogram plots show fluorescence at the FL1-A channel. Results were represented as a percentage of the cell population with internalized bacteria. **C.** Fluorescence microscopy showing the degree of bacterial association of live and dead bacilli after 48 h of incubation with SC at 33°C, MOI 50:1. BCG WT, BCG PGL I, BCG PGL TB, and *M leprae* were labeled with green fluorescent PKH67. Nuclei were stained with DAPI (blue). Scale 10μm. Quantification of the percentage of cells with associated bacteria in 200 fields per condition and per replicate. **D.** Primary human Schwann (PHSC) cells were treated with BCG WT, BCG PGL TB, BCG PGL I or *M*. *leprae* for 48 h at 33°C, MOI 50:1. Bacteria were previously labeled with PKH67; and the degree of internalization was determined after Trypan Blue quenching. Percentage of cells with internalized bacteria was determined using flow cytometry. **E.** PHSC were treated with green fluorescent latex beads covered or not with PGL I. The percentage of cells with internalized beads was determined after 48 h of incubation at 33°C and a 50:1 proportion via flow cytometry. Each bar represents the mean ± SEM from at least three independent experiments in triplicate. An ANOVA test followed by Bonferroni as a post-test were performed and used for statistical analyses. ** p<0.01; ***p<0.001. In E) Statistical significance was calculated by Mann Whitney Test **p < 0.01.

As a first step, the critical role of PGL I on *M*. *leprae* adhesion and SC internalization was addressed by comparing the capacity of the recombinants BCG PGL I, BCG PGL TB—a recombinant BCG strain that produces PGL from *M*. *tuberculosis* (BCG PGL TB) instead of PGL bovis [25]—and wild type BCG (BCG WT), their parental strain, to infect the human schwannoma cell line ST8814. The recombinant bacteria were labeled with the green fluorescent dye PKH67; and association (%) was determined after 4 h, 24 h and 48 h of incubation with SC at MOI 50:1 by flow cytometry. Association (adhesion + internalization) values showed that the PGL I-expressing recombinant strain acquired a high capacity to interact with these cells as opposed to the lesser capacity of the BCG WT strain (histogram plot-Fig 2A). Interestingly, BCG PGL TB behaved similarly to the wild-type strain, indicating that the observed effect was mediated by the unique trissacharide moiety of *M*. *leprae* PGL (Fig 2A).

For BCG PGL I, a time-dependent association between the bacterium and SCs was observed, with about 80% of cells evidencing associated bacilli after 48 h of incubation (Fig 2A). At this time point, most of the bacilli had been internalized, as monitored by quenching extracellular bacteria with Trypan Blue (S1A Fig). Moreover, S1B Fig delineates that, in assays performed at 4°C to inhibit internalization, PGL I was shown to mediate the adhesion step. We also made use of GFP expressing recombinant BCG PGL I or PGL TB and the parental BCG strain to confirm their differential capacity to associate to SCs (S1C Fig).

Since host cell internalization could be an active process dependent on bacterial viability, we next compared the ability of live versus lethally-irradiated bacterial cells to invade SCs (Fig 2B). For this purpose, the degree of internalization was determined by quenching the fluorescence of adhered bacteria with Trypan Blue (Fig 2B, S2A Fig). *M*. *leprae* was included in these assays with results equivalent to those obtained with BCG PGL I. Both dead BCG PGL I and dead *M*. *leprae* measured roughly 50% less internalization capacity in contrast to live BCG PGL I and live *M*. *leprae*, respectively (dead BCG PGL I 30.33 ± 5.21% and dead *M*. *leprae* 40.66 ± 4.41%). S2B Fig shows that the percentage of SCs with internalized bacteria augmented in accordance with an increasing multiplicity of infection (MOI). In comparing dead with live bacilli at a MOI of 10:1 and 50:1, it was seen that when dead bacteria were used the SC population with internalized bacteria fell about 50%. Interestingly, however, at a MOI of 100:1, both live and dead bacilli were present in nearly 100% of the cell population. Nevertheless, regardless of the MOI used, in analyzing the Median Intensity of Fluorescence (MFI) of the cell population, the use of live bacteria resulted in significantly higher values, indicating that the number of bacilli per cell was always higher in the ones infected with live bacilli (S2C Fig).

The importance of PGL I in SC invasion was confirmed by fluorescence microscopy (Fig 2C). After 48 h of infection, the percentage of ST8814 cells with associated BCG PGL I was more elevated (88.33 ± 3.79%) than that found for either BCG WT (10.17 ± 2.47%) or BCG PGL TB (10.50 ± 1.00%). *M*. *leprae* was used as a positive control, resulting in the presence of associated bacteria in 88.67 ± 3.51% of the cells. Also, the influence of viability on SC invasion was corroborated since the association capacity of dead BCG PGL I and dead *M*. *leprae* was about 50% less in comparison to live BCG PGL I and live *M*. *leprae*, respectively (dead BCG PGL I 41.00 ± 6.00% and dead *M*. *leprae* 47.33 ± 2.52%) while, when compared to live mycobacteria, dead BCG WT and BCG PGL TB maintained the same levels.

Since ST8814 is a tumor cell line, similar experiments were repeated with Primary Human Schwann Cells (PHSC). As shown in Fig 2D, the critical role played by PGL I in the bacterial SC invasion was validated in that the association values attained by BCG PGL I were similar to those for *M*. *leprae* itself. Likewise, as seen with ST8814 cells, the internalization of live bacteria was significantly higher than that found for dead bacilli in the context of both BCG PGL I and *M*. *leprae*. Moreover, PGL I-covered beads showed a higher internalization rate than uncovered ones (Fig 2E), attesting to the importance of PGL I in mycobacterium SC internalization. Altogether, these results confirm that the unique phenolic glycolipid produced by *M*. *leprae* is a key molecule involved in bacterial adhesion and SC internalization and that bacterial viability strongly facilitates entry.

### Pre-infection with live PGL I-producing mycobacteria allows for BCG WT Schwann cell internalization

**Fig 3:**
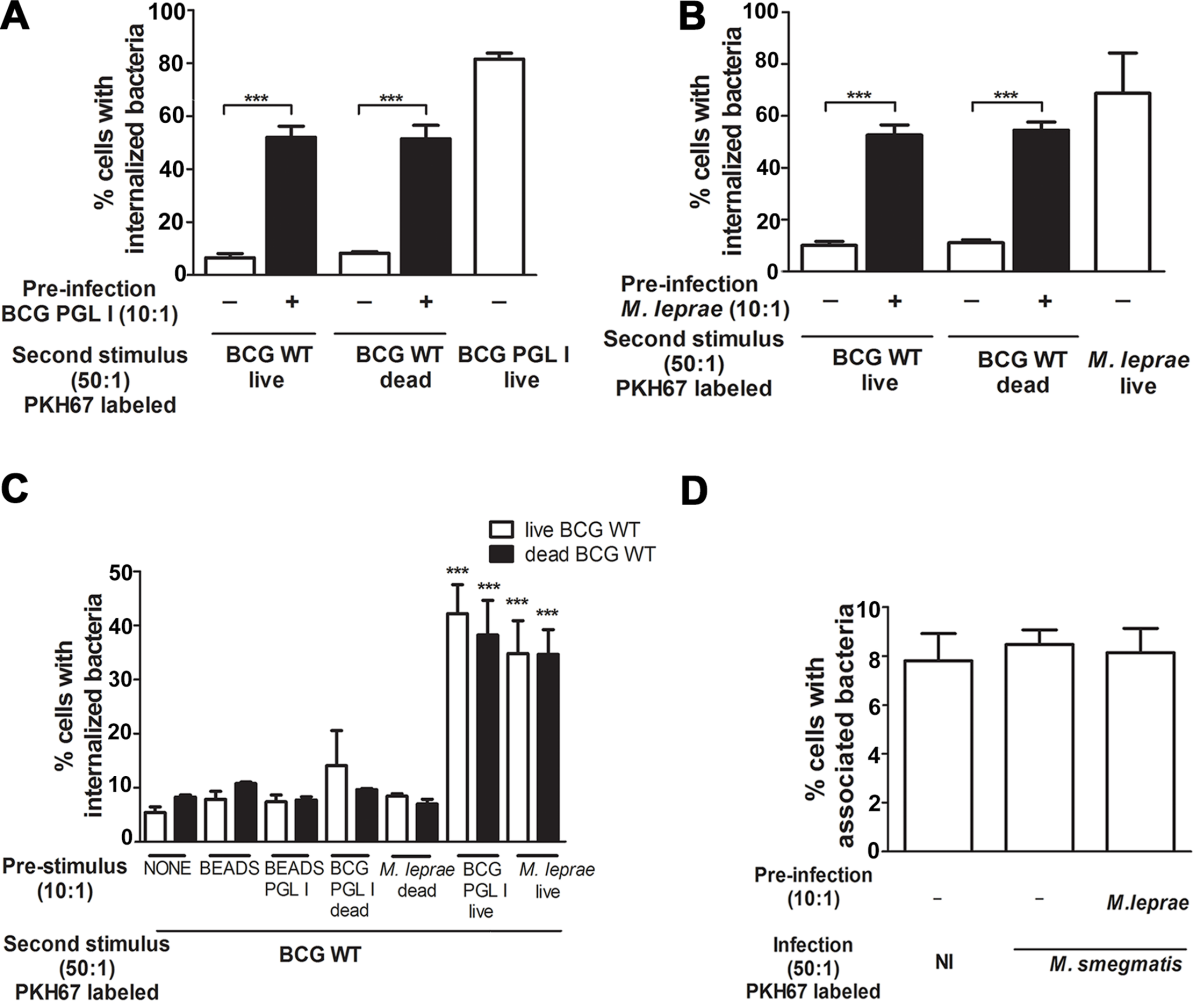
Pre-infection with live BCG PGL I or *M*. *leprae* allows BCG WT to enter Schwann cells. **A-B.** A higher degree of association and internalization of PKH67-labeled BCG WT (live or irradiated) was observed after pre-infection with live BCG PGL I or *M*. *leprae* at MOI 10:1. **C.** A first stimulus with beads, beads covered with PGL I, and dead *M*. *leprae* or dead BCG PLG I did not result in a higher degree of BCG WT internalization. **D.** Flow cytometric results showed no change in the degree of internalization of PKH67-labeled *M*. *smegmatis* after pre-stimulus with *M*. *leprae* at MOI 10:1. (NI shows background fluorescence). **A-D.** Results are represented as mean ± SEM of at least three independent biological replicates; and statistical significance was calculated by ANOVA followed by Bonferroni’s multiple comparison test. *** p<0.001.

Intracellular pathogens are known to modulate host-cell phagocytic receptors as a way to facilitate their entrance into host cells. We, therefore, turned to investigating whether *M*. *leprae* or BCG PGL I modulate the SC phagocytic phenotype. A series of experiments using different combinations of pre- and secondary stimuli were conducted with ST8814 SC. The first stimulus consisted of unlabeled bacteria or latex beads in a 10:1 proportion. The second stimulus with a 50:1 proportion consisted of the addition of PKH67 (green)-labeled bacteria or green fluorescent latex beads 1 hour after the initial stimulus. Cells were then incubated for 48 h and analyzed via flow cytometry or fluorescence microscopy. Surprisingly, pre-infection with BCG PGL I or *M*. *leprae* led to a significant internalization of BCG WT (live or dead) in SCs (Fig 3A and 3B).

Contrariwise, dead *M*. *leprae* and BCG PGL I were unable to instigate a major upgrade in SC internalization (Fig 3C). PGL I-covered latex beads were likewise unable to mimic the effect of PGL I-expressing bacteria (Fig 3C). Alternatively, even the direct addition of purified PGL I to the medium had no tangible effect on the SC phagocytic response (S3A Fig). Moreover, the expanded phagocytic capacity induced by *M*. *leprae* and BCG PGL I on SC was specific to BCG WT since the degree of internalization of non-pathogenic *M*. *smegmatis* (Fig 3D) and the uptake of green fluorescent latex beads remained unchanged (S3B Fig). To summarize, these results suggest that live PGL I-producing mycobacteria modulate the expression in SCs of phagocytic receptors that recognize cell wall envelope components specifically expressed by pathogenic or slow growing mycobacteria.

### Infection with PGL I-producing mycobacteria upregulates CD206 expression in Schwann cells

LAM, present in the mycobacterial cell envelope, is an abundant glycolipid shown to play a key role in mycobacteria-host cell interaction [26, 27]. In pathogenic mycobacteria, this molecule, denominated ManLAM [28], is mannose capped. It has been demonstrated that the terminal mannose residues of ManLAM are of critical importance in macrophage recognition of *M*. *tuberculosis*, *M*. *leprae* and *M*. *bovis* BCG [29, 30] via the capacity of these residues to bind to the host cell mannose receptor (MR/CD206) [31]. In contrast, *M*. *smegmatis* produces a structurally different LAM that is capped with phosphatidyl-myo-inositol residues that do not bind to CD206 [32].

**Fig 4:**
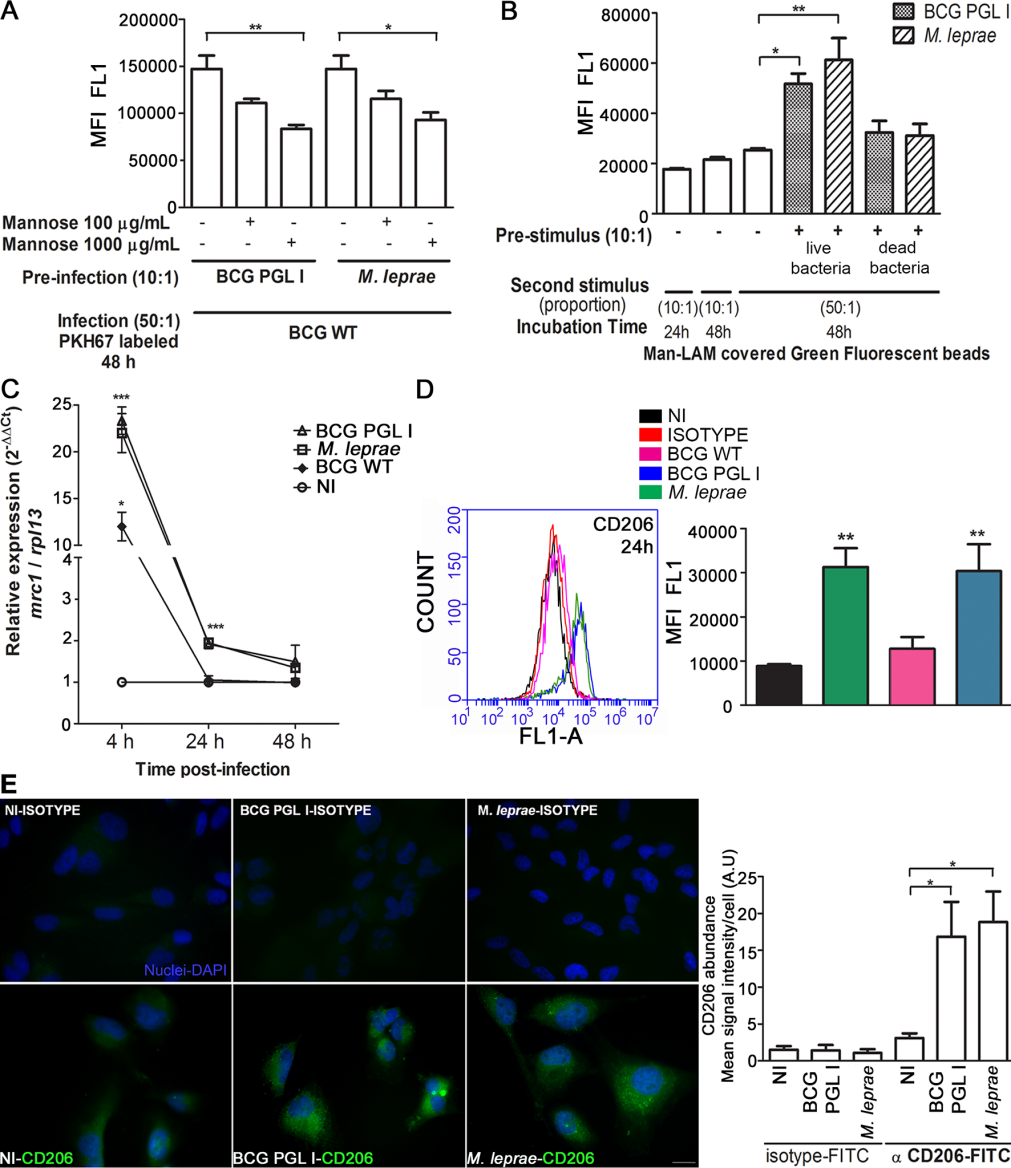
Infection with BCG PGL I or *M*. *leprae* induces CD206 expression in Schwann cells. **A.** Competition assay suggesting the Mannose Receptor (CD206) as a receptor candidate to mediate the internalization of BCG WT in SC. After pre-infection with *M*. *leprae* or BCG PGL I, the addition of mannose at 100 or 1000 μg/mL reduced the BCG WT internalization rate 48 h post-infection. In all experiments, the degree of internalization of PKH67-labeled BCG WT was determined after Trypan Blue quenching by flow cytometry. **B.** Pre-infection with PGLI-expressing bacteria MOI 10:1 favors the internalization of ManLAM-covered latex beads, which were incubated with SC for 48 h at 33°C and a 50:1 proportion. MFI was determined using the flow cytometry FL1-A channel. Green fluorescent latex beads covered with ManLAM showed a higher degree of internalization in comparison to the control green fluorescent latex beads. **C.** Normalized relative expression of *mrc1* (delta delta Ct) in SC infected for 4 h, 24 h or 48 h with either BCG PGL I, *M*. *leprae*, or BCG WT at MOI 50:1. Results are presented in terms of fold changes after normalization with *rpl*13 mRNA. **D.** CD206 expression in SC after 24 h of infection was measured by flow cytometry. Immunofluorescent labeling of CD206 was carried out using the FITC conjugated anti-CD206 antibody. The conditions were: uninfected (NI), isotype control, and treatment with BCG WT, BCG PGL I, or *M*. *leprae*. A representative histogram plot shows fluorescence at the FL1-A channel. **E.** Representative images of fluorescence microscopy showing CD206 expression in uninfected SC and *M*. *leprae* or BCG PGL I-infected SC after 24 h of incubation. Cells on coverslips were fixed with paraformaldehyde and stained with DAPI (blue) for nuclear localization. Cells were immunolabeled with FITC conjugated anti-CD206 antibody or a FITC conjugated isotype and then examined by fluorescence microscopy. The mean CD206 signal intensity per cell was quantified. Scale bar: 10μm (white line) **A-E.** Results are represented as mean ± SEM of three or more independent biological replicates. Statistical significance was calculated by ANOVA followed by Bonferroni’s multiple comparison test. *p < 0.05; ** p<0.01; *** p<0.001.

Based on this knowledge, we postulated the candidacy of the mannose receptor as possible target of upregulation by PGL I-expressing mycobacteria in infected SCs. To validate this hypothesis, the effect of excess free mannose on BCG WT internalization in SCs pre-infected with *M*. *leprae* or BCG PGL I by flow cytometry was evaluated. This competitive assay showed that the presence of mannose at 100 μg/mL or 1000 μg/mL significantly reduced BCG WT internalization induced by pre-infection with BCG PGL I or *M*. *leprae* (Fig 4A). Under the condition of 1000 μg/mL of mannose, the degree of BCG WT internalization decreased by about 40%. Fluorescence microscopy also showed decrease in association degree (S4 Fig), suggesting the recognition of LAM mannose caps during BCG WT internalization. To further explore this idea, green fluorescent latex beads were covered with *M*. *leprae*-purified ManLAM and used as the second stimulus in SCs pre-infected with live BCG PGL I or *M*. *leprae*. ManLAM-covered beads showed an increased uptake as seen for BCG WT, pointing to the upregulation of a mannose receptor in pre-infected cells (Fig 4B).

Since CD206 was previously shown to be expressed by SCs [33, 34], we then explored the ability of *M*. *leprae* or BCG PGL I to modulate this receptor. The *mrc1* gene transcriptional levels in SCs were assayed at different time points by quantitative RT-PCR. Fig 4C shows the relative expressions of normalized *mrc1* (delta delta Ct) in BCG PGL I-, *M*. *leprae*-, or BCG WT-infected SCs at 4 h, 24 h and 48 h. Results are presented in terms of a fold change after normalization with ribosomal protein L13 (RPL13) mRNA. It was found that *M*. *leprae* and BCG PGL I were capable of inducing *mrc1* transcription in SCs. It was also seen that the upregulation of *mrc1* occurred at an early stage in the infection in that, a mere 4 h later, the transcription level had reached a peak. Of note, BCG WT also induced *mrc1* gene upregulation but at a 50%-or-less efficacy rate as compared to the bacilli-expressing PGL I. It was, however, solely in the presence of *M*. *leprae* or BCG PGL I that upregulation was sustained until 24 h post-infection (Fig 4C). Accordingly, flow cytometry assays showed an increased expression of CD206 upon *M*. *leprae* or BCG PGL I but not BCG WT infection (Fig 4D). Comparable results were observed by fluorescence microscopy analysis (Fig 4E), indicating that the expression of CD206 is upregulated in SCs infected with PGL I-expressing bacilli.

**Fig 5:**
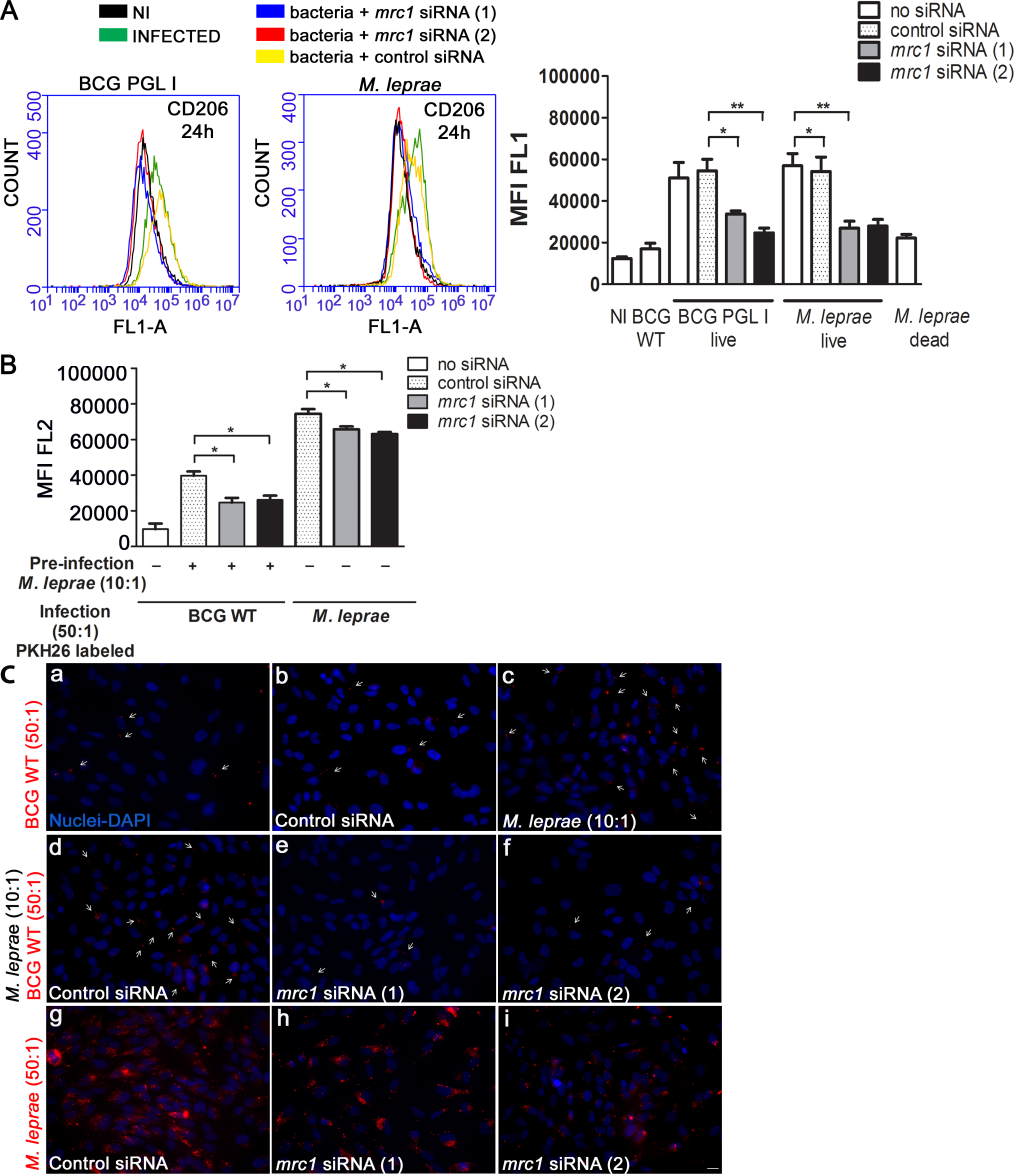
Mannose receptor *mrc1* knockdown diminishes BCG WT and *M*. *leprae* entry into Schwann cells. **A**. Verification of *mrc1* gene knockdown. SC were transfected with control siRNA or two siRNA targeting *mrc1* for 24 h before infection. Flow cytometry result showing that knockdown with *mrc1*siRNA reduces CD206 expression in *M*. *leprae*-infected SC. Representative histogram plots show fluorescence at the FL1-A channel. SCs were submitted to immunofluorescent labeling of CD206 with the FITC conjugated anti-CD206 antibody (clone15-2, Mouse IgG1, κ1, Biolegend). SC expression of CD206 after 24 h of infection at MOI 50:1, 33°C was measured using the flow cytometry FL1-A (green) channel to determine MFI. **B**. Flow cytometry result showing the effect of *mrc1* knockdown on the degree of association of PKH26-(red) labeled *M*. *leprae* or BCG WT. For BCG WT, the previously described co-infection assay was applied. MFI was determined at the FL2-A channel. **C**. SC on coverslips were stimulated with *M*. *leprae* or BCG WT for 48 h. The cells were fixed with paraformaldehyde, stained with DAPI (blue) for nuclear localization, and examined by fluorescence microscopy. The upper panel shows (***a-b***) BCG WT association with and without control siRNA, (***c***) co-infected SC with *M*. *leprae* and PKH26-labeled BCG WT. The middle panel shows (***d***) co-infection in the presence of control siRNA, (***e-f***) co-infection in the presence of two types of *mrc1* siRNA. The lower panel shows PKH26-labeled *M*. *leprae* associated to SC in the presence of (***g***) control siRNA, (***h- i***) tow types of *mrc1* siRNA. Scale (white line) represents 10 μm. Results are represented as mean ± SEM of three independent biological replicates. Statistical significance was calculated by ANOVA followed by Bonferroni’s multiple comparison test. *p < 0.05; **p<0.01; ***p<0.001.

That *M*. *leprae* and BCG PGL I infection led to the upregulation of CD206 and its subsequent involvement in the uptake of BCG WT was confirmed by *mrc1* knockdown experiments. Treatment with *mrc1* siRNA targeting the *mrc1* gene coding for CD206, led to a significant reduction in CD206 expression in BCG PGL I and *M*. *leprae*-infected SC, as compared to cells transfected with the control siRNA (Fig 5A). As monitored by flow cytometry (Fig 5B) and fluorescence microscopy (Fig 5C), *mrc1* knockdown resulted in a significant reduction in the degree of BCG WT internalization. Of note, *mrc1* knockdown also decreased *M*. *leprae* internalization (Fig 5B and 5C). Taken together, these results show that live PGL I-producing mycobacteria induce the expression of the mannose receptor CD206 in SCs, a mechanism that may promote *M*. *leprae* internalization by an alternative pathway.

### CD206 and PPARγ upregulate each other in Schwann cells infected with PGL I-producing mycobacteria

Previous reports have shown a reciprocal regulation between CD206 and the transcription factor peroxisome proliferator-activated receptor gamma (PPARγ) [35, 36]. Furthermore, the induction and activation of PPARγ by pathogenic mycobacteria following macrophage infection has been linked to their capacity to persist in these cells [34, 36, 37].

**Fig 6:**
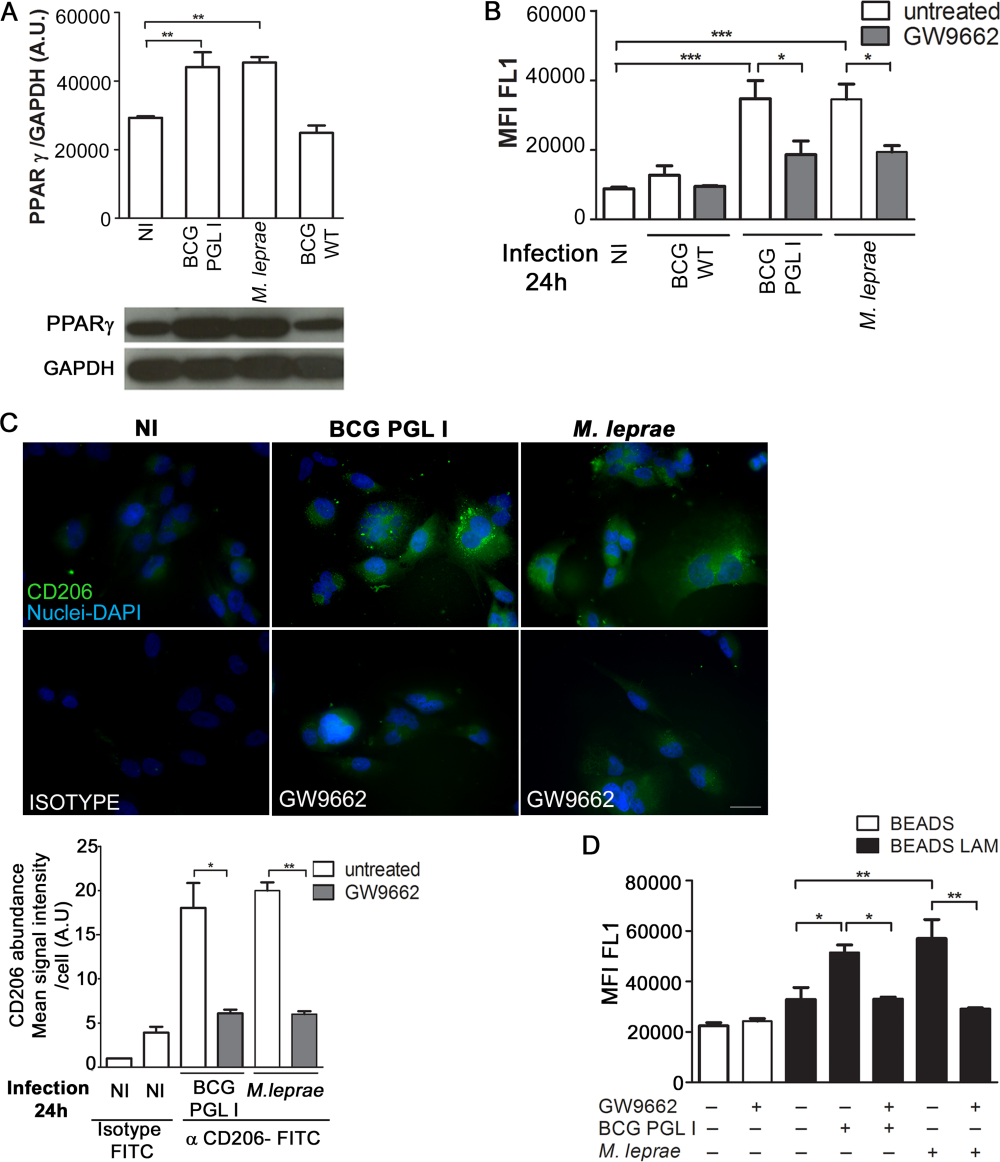
PGL I-producing mycobacterium induces CD206 expression in Schwann cells via PPARγ. **A.** Total lysates (20μg per well) from SC cultures were subjected to Western Blotting using specific antibodies against PPARγ and GAPDH. A representative Western blot from three independent experiments is shown. The content of the bands was estimated by densitometric analysis; and relative expression was plotted in arbitrary units (A.U. = arbitrary units). **B and C.** SC were treated with the PPARγ antagonist GW9662 (5μM) for 30 minutes previous to a 24 h infection with either BCG WT, BCG PGL I, or *M*. *leprae* at MOI 50:1. CD206 expression was determined using flow cytometry and fluorescence microscopy by immunolabeling of CD206 with FITC conjugated anti-CD206 antibody (clone15-2, Mouse IgG1, κ1, Biolegend). Treatment with GW9662 decreased CD206 expression in SC. For microscopy, cells on coverslips were fixed with paraformaldehyde and stained with DAPI (blue) for nuclear localization. Images are representative of 3 independent experiments. Scale (white line) represents 10 μm. CD206 mean signal intensity per cell was quantified. **D.** SC were treated with GW9662 5μM for 30 minutes previous to 48 h stimulation with either uncovered beads or ManLAM-covered beads at a proportion of (50:1). Pre-stimulus with unlabeled *M*. *leprae* or BCG PGL I was carried out after treatment with the antagonist and one hour before the second stimulus. Internalization of the green fluorescent beads was determined by flow cytometry (FL1-A channel). Results are represented as mean ± SEM of three independent biological replicates. Statistical significance was calculated by ANOVA followed by Bonferroni’s multiple comparison test. *p < 0.05; **p<0.01; ***p<0.001.

We thus analyzed the potential involvement of PPARγ in inducing CD206 in *M*. *leprae* and BCG PGL I-infected SCs. Fig 6A indicates that *M*. *leprae* and BCG PGL I infection induce PPARγ expression in contrast to BCG WT, which does not. Moreover, cells pre-treated with GW9662, an irreversible antagonist of PPARγ, showed reduced CD206 levels in response to *M*. *leprae* and BCG PGL I monitored 24 h post-infection by both flow cytometry (Fig 6B) and fluorescence microscopy (Fig 6C). At this time point, the percentage of SCs with internalized bacteria remained unchanged in GW9662-treated cells as compared to untreated ones (S5A Fig). However, after 48 h of infection, GW9662 pre-treated cells demonstrated a lower number of *M*. *leprae* bacilli per cell, comparable to that observed after the *mrc1* knockdown (S5B Fig). Additionally, cell pre-treatment with GW9662 combined with pre-infection with PGL I -expressing mycobacteria were unable to increase ManLAM-covered bead uptake (Fig 6D), confirming the involvement of PPARγ in the upregulation of CD206 in SCs infected with mycobacteria expressing PGL I.

**Fig 7:**
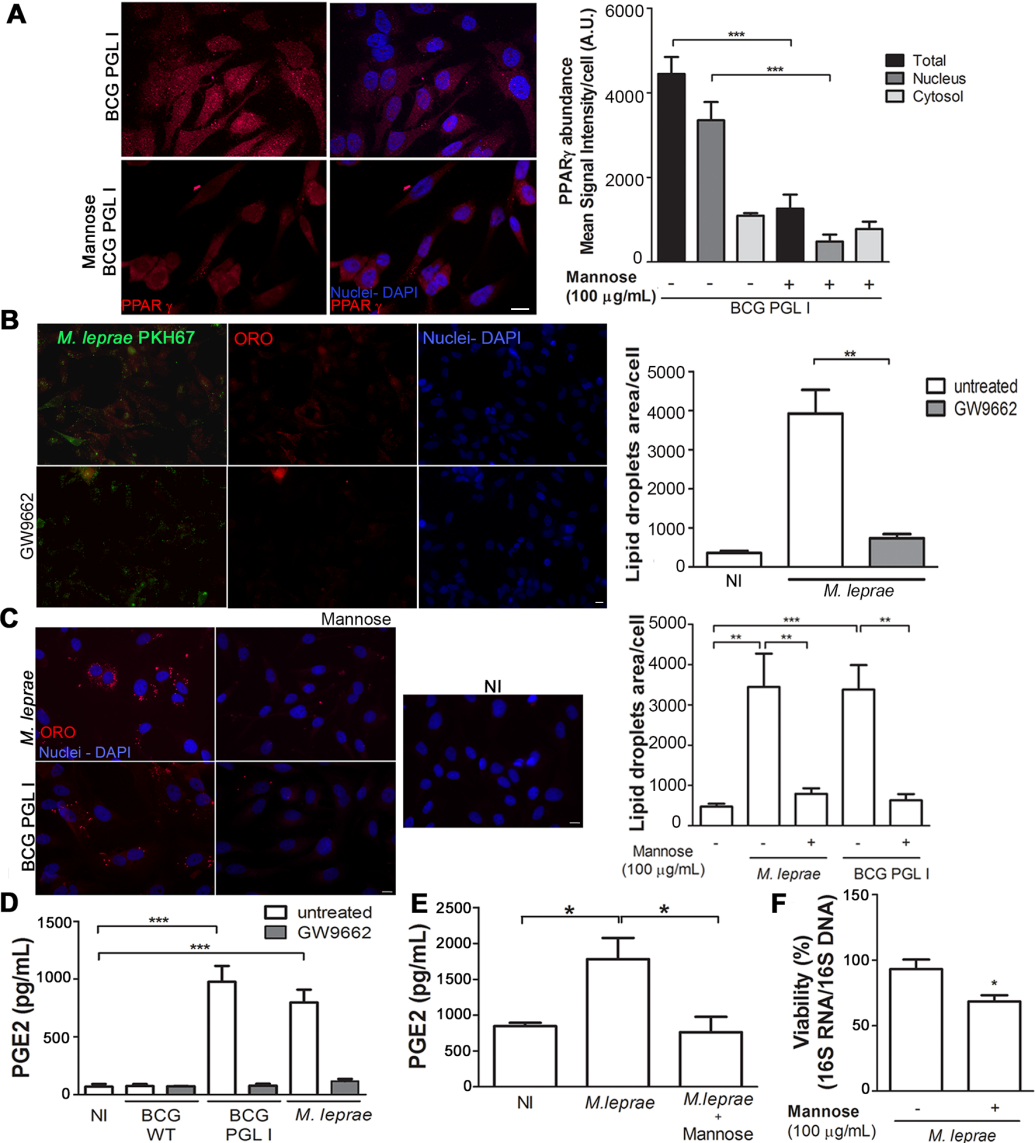
Crosstalk between PPARγ and CD206 mediates lipid droplet and PGE2 production in Schwann cells infected with PGL I-producing mycobacteria. **A.** Competition assay suggesting cross-talk between PPARγ and CD206 in SCs. The addition of mannose at 100 μg/mL reduced BCG PGL I-induced PPARγ expression 48 h post-infection. PPARγ detection was performed using the specific rabbit polyclonal antibody (H-100) SC-7196 (Santa Cruz Inc., USA) followed by incubation with IgG anti-rabbit conjugated to Alexa Fluor 594 (Molecular Probes, USA) for immunofluorescence detection. Cells on coverslips were fixed with paraformaldehyde and stained with DAPI (blue) for nuclear localization. Representative fluorescence microscopy images showing the expression and localization of PPARγ after addition of mannose. Mean signal intensity per cell was quantified. Scale bar: 10μm (white line). **B and C.** Impact of the transcription factor PPARγ and the CD206 receptor on LD formation induced by *M*.*leprae* and BCG PGL I in SCs. Representative fluorescence microscopy images showing the effect of PPARγ antagonist GW9662 (5 μM) (B) and of mannose 100 μg/mL (C) on LD induction. The LD induction was estimated by microscopy after fluorescent Oil Red O staining and quantification of ORO-stained LDs was plotted as measurement of LD area/cell. The pretreatment with GW9662 or mannose respectively, reduced the LD formation induced by *M*.*leprae* or BCG PGL I 48 h post-infection. Scale bar: (A) 20μm (B) 10 μm and (C) 20 μm (white line). Data are shown as mean±SEM of three (7B) and five (7C) different experiments performed in triplicate. **D and E**. EIA was used to analyze the effect of the addition of GW9962 or mannose on the levels of PGE2 in the supernatants from the LD induction experiments. The results are the mean ± SEM from at least three independent experiments performed in triplicate. Statistical significance was calculated by ANOVA followed by Bonferroni’s multiple comparison test. *p < 0.05; ** p<0.01; ***p<0.001. **F**. *M*. *leprae* viability measured by qRT-PCR using the ratio of 16S rRNA/16S DNA 48 h after infection of ST8814 pre-treated or not with 100 μg/mL mannose. Data are shown as mean±SEM of six different experiments performed in triplicate. Statistical significance was calculated by Mann Whitney Test *p < 0.05.

An evaluation was then carried out to determine whether bacterial recognition via CD206 was involved in PPARγ induction and activation. To test this hypothesis, live bacteria were incubated with cells in the presence of an excess of free mannose to block bacterial recognition by CD206. The induction and activation of PPARγ was monitored via immunofluorescence. As shown in Fig 7A, significantly lower levels of PPARγ induction and activation were observed in the presence of free mannose, suggesting the involvement of bacterial recognition via CD206 in the capacity of *M*. *leprae* to activate this nuclear receptor/transcriptional factor in SCs.

### Crosstalk between PPARγ and CD206 mediates LD biogenesis and PGE2 production in Schwann cells infected with PGL I-producing mycobacteria

Since PPARγ has been implicated in LD biogenesis induced by mycobacteria [37], we next evaluated whether this was the case in the context of *M*. *leprae* infected SCs. Inhibition of PPARγ with GW9662 abolished the induction of LDs (Fig 7B) by *M*. *leprae*. Moreover, inhibiting signaling from MR/CD206 by infecting the cells in the presence of excess of free mannose reduced LDs levels significantly (Fig 7C). In previous reports we showed that *M*. *leprae-* induced LDs constitute sites of (PGE2) synthesis [22]. Next, we investigated the role of MR/PPARγ crosstalk in PGE2 production. PPARγ inhibition decreased PGE2 production to baseline levels in response to *M*. *leprae* (Fig 7D). A similar effect was observed when cells were infected in the presence of excess of free mannose (Fig 7E). Experiments were then conducted to monitor the effect of *mrc1* knockdown on PGE2 production in response to *M*. *leprae*. However, the high background of PGE2 production in the uninfected cells transfected with the *mrc1* siRNA prevented reaching a reliable conclusion (S6 Fig).

It was then determined to examine if the recognition of *M*. *leprae* by CD206 causes an effect on subsequent bacterial intracellular survival since LDs formation was previously shown to promote *M*. *leprae* persistence in infected cells [23]. To block recognition of *M*. *leprae* by CD206, an excess of free mannose (100 μg/mL) was added to SCs followed by the monitoring of bacterial viability. Fig 7F shows a 27% (*p<0.05) decrease in cellular bacterial viability upon 48 h of infection when bacterial recognition by CD206 was inhibited. These results provide evidence that CD206-PPARγ crosstalk promotes bacterial survival in *M*. *leprae*-infected SCs.

**Fig 8:**
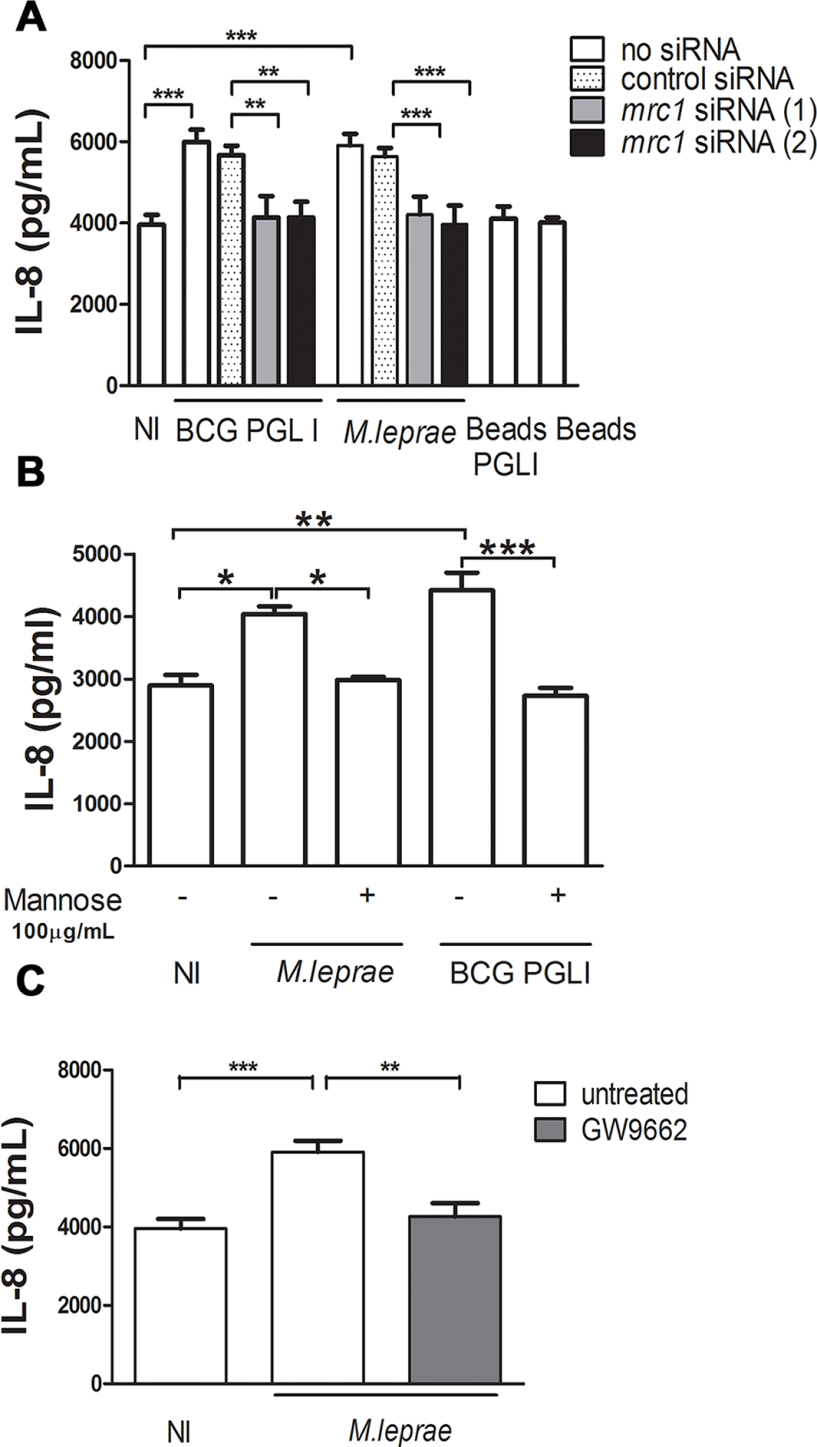
Crosstalk between PPARγ and CD206 mediates IL-8 production in Schwann cells infected with PGL I-producing mycobacteria. **A.** SCs were transfected for 24 h with control siRNA or siRNA targeting *mrc1*, followed by infection with *M*. *leprae* or BCG PGL I for 48 h. Supernatants were analyzed for IL-8 production by ELISA. **B and C.** Alternatively, cells were pretreated with mannose 100 μg/mL or the PPARγ antagonist GW9662 (5 μM) respectively, 30 minutes before infection. Cells were then infected with *M*. *leprae* or BCG PGL I for 48 h. Supernatants were analyzed for IL-8. The results are the mean ± SEM from at least three independent experiments performed in triplicate. Statistical significance was calculated by ANOVA followed by Bonferroni’s multiple comparison test. *p < 0.05; ** p<0.01; ***p<0.001.

The connection of CD206 to PPARγ and its role as an important regulator of macrophage immune response to *M*. *tuberculosis* has recently been reported [35, 38]. In that study, a signaling pathway involving recognition of Man-LAM by CD206 followed by PPARγ expression and activation terminating in IL-8 induction was described [35]. In this context, our next step involved examining whether this pathway was active in mycobacterium-infected SCs. To answer this question, the effect of *mrc1* knockdown or inhibition of PPARγ activity on IL-8 was investigated. Our results demonstrated that *M*. *leprae* infection enhanced IL-8 production and that knocking down *mrc1* caused a decrease on IL-8 production (Fig 8A). Moreover, infection in the presence of an excess of free mannose evoked a similar inhibitory effect of IL-8 secretion (Fig 8B). Also, treatment with GW9662 decreased IL-8 production indicating the involvement of PPARγ (Fig 8C). Altogether, these results suggest that there is an active crosstalk between PPARγ and CD206 that links lipid metabolism with the downstream innate immune response in SCs infected with PGL I-producing mycobacteria.

### CD206 is expressed by SCs in *M*. *leprae* infected nerve lesions

**Fig 9:**
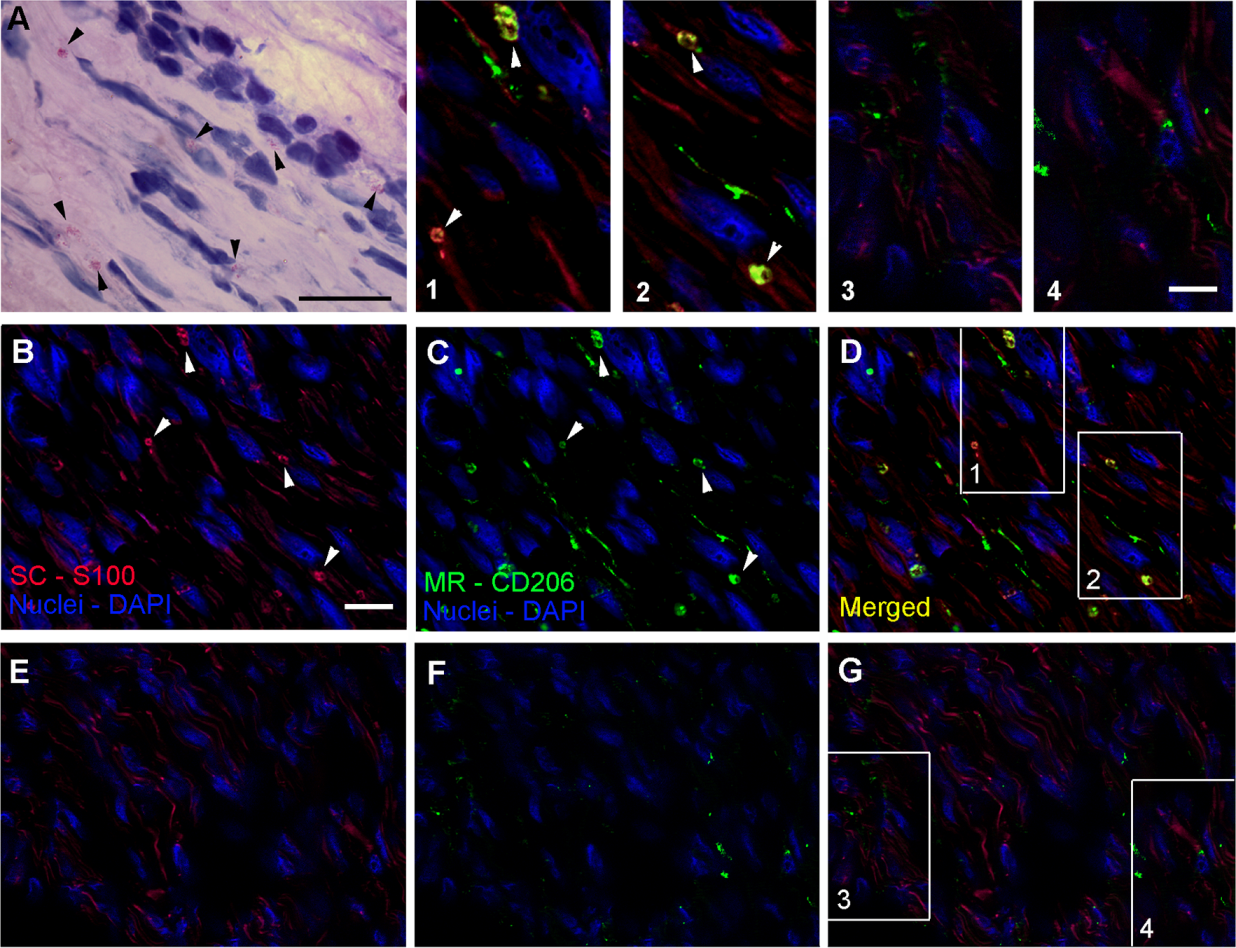
CD206 colocalizes with Schwann cells in nerve lesions of leprosy patients. Nerve biopsies were labeled with antibodies for the SC-specific marker S100 (red image), and the mannose receptor CD206 (green image) and then visualized by fluorescence microscopy. Nuclei were labeled with DAPI (blue image). **A-D.** Serial sections of a leprosy patient nerve biopsies were analyzed. **A.** Wade staining showing *M*.*leprae*. Scale bars, 20μm. **B-D**. Tissue sections were visualized by fluorescence microscopy showing CD206/S100 co-staining SCs. Images are representative of a total of five patients. **E-G.** Serial sections of a nerve biopsy from a patient with a non-leprosy peripheral neuropathy. The merged image in **G.** shows no CD206/S100 co-localization.Images are representative of a total of three patients. Scale bar, 20μm. Insets: magnified views of CD206/S100 staining in leprosy (insets 1 and 2) and non-leprosy (insets 3 and 4) nerve biopsies. Scale bar, 10μm.

**Fig 10:**
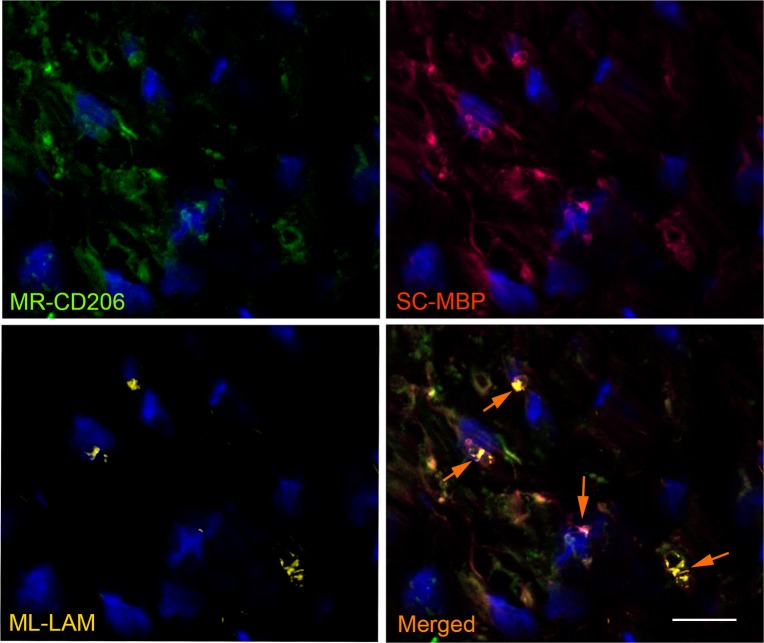
*M*. *leprae* infected Schwann cells express CD206 in leprosy nerve lesions. Nerves biopsies were labeled with antibodies for mannose receptor CD206 (green image), for the SC-specific marker MBP (red image) and for *M*.*leprae* (anti-LAM; yellow image). Nuclei were labeled with DAPI (blue). The serial section of a leprosy patient nerve biopsy was analyzed by fluorescence microscopy. The images (representative of two patients) show the expression profile of the CD206 mannose receptor, the MBP SC marker and the location of *M*. *leprae*. The merged image shows CD206/MBP/LAM co-staining in SCs (white arrows). Scale bar, 20μm.

The ability of *M*. *leprae* to induce the expression of CD206 in SCs cultures prompted us to further examine leprosy nerve specimens to corroborate the *in vitro* findings with *in situ* evidence. To verify if CD206 was expressed by SCs, tissue sections of patients with leprosy and non-leprosy neuropathies were also stained with anti-S100, a specific SC marker (Fig 9, S7 Fig, S8 Fig). Nerve biopsies previously known to be bacilli positive (AFB^(+)^) were chosen for this analysis, as shown by Wade's [39] staining in Fig 9A. Fig 9B and 9C show the staining profile of S100 and the mannose receptor CD206, respectively. Cells with a SC morphology expressing CD206 and S100 were observed in leprosy patients (Fig 9D). CD206-expressing SCs can be better visualized in the insets (1, 2) with a magnified view. Nerve biopsies from other 4 patients were analyzed generating similar results (S8 Fig). Nerve biopsies obtained from patients with non-leprosy neuropathies showed no CD206/S100 colocalization (Fig 9E–9G (insets 3 and 4), S7 Fig). In order to confirm that SCs expressing CD206 were harboring *M*. *leprae*, leprosy nerve tissue sections were labeled with anti-CD206, anti-Myelin Basic Protein (MBP), a SC marker, and anti-Liporabinomannan (LAM) for mycobacterium staining. Fig 10 shows most cells in the field double stained for CD206 and MBP, confirming the presence of SCs expressing CD206 in leprosy patients nerves. Since one Schwann cell may spread over hundreds of μm long nerve, a serial slices analysis would probably be necessary to confirm the presence of bacteria in a single cell. Nevertheless, examining a single cross-sectioned slice of the nerve biopsy we were able to detect *M*. *leprae* inside half of the SCs. Altogether, these data strongly suggest that *M*. *leprae* induces the expression of CD206 in *in vivo*-infected SCs reproducing our *in vitro* findings.

## Discussion

The most severe symptoms of leprosy are caused by nerve infection. Thus, deciphering the molecular basis of the early events of mycobacterial peripheral nerve infection is a crucial step towards acquiring a basic understanding of nerve pathogenesis with the potential to generate new tools for its prevention and treatment. The capacity of *M*. *leprae* to bind to laminin-2, a major component of the SC basal lamina, has been described as a fundamental feature of the bacterial predilection for the peripheral nervous system [6]. Moreover, the PGL I and Hlp/LBP-21 molecules produced by the leprosy bacillus located on the bacterial surface have been implicated as likely adhesins involved in this interaction [12].

In the present study, we revisited the involvement of PGL I in *M*. *leprae*-SC interactions by using a recombinant strain of *M*. *bovis* BCG engineered to express PGL I. We demonstrate for the first time that PGL I is essential for mycobacterium adhesion and SC internalization. We were also able to confirm that the unique trisaccharide moiety of PGL I mediates the specific *M*. *leprae*-SC interaction since other phenolic glycolipids with identical lipid moieties proved incapable of mediating bacterial internalization into these cells. Of note, it was found that live PGL I-producing mycobacterium induces the activation of a crosstalk between the endocytic receptor MR/CD206 and the transcriptional factor PPARγ, allowing bacterial recognition and entry by way of this alternative pathway. Bacterial sensing via mannose recognition was shown to be essential for the induction of LD formation, organelles previously shown to play a key role in *M*. *leprae*-SC interaction [22, 23]. Finally, the detection of strongly positive CD206 SCs in leprosy nerve sections suggests that *M*. *leprae* induces CD206 expression in *in vivo*-infected SCs, which may be critical in the development of bacterial pathogenesis in the nerve.

The results herein presented firmly indicate that PGL I is the key molecule responsible for capacitating *M*. *leprae* to successfully invade SCs. In this process, PGL I imposes a secondary role on other potential adhesins such as Hlp/LBP-21, a well-conserved, histone-like protein present on the surface of several species of mycobacteria, including BCG [19]. Recombinant Hlp binds *in vitro* to the laminin-2 globular domain; and latex beads covered with the protein have been seen to display a greater capacity to adhere to and be internalized by SC [10, 11]. Indeed, other species of mycobacteria were shown to bind laminin-2 and adhere to SCs in the presence of soluble alpha2-laminin [40]. However, most evidence suggesting a role for Hlp in *M*. *leprae*–SC interactions have been drawn from assays conducted with the isolated protein alone in the absence of PGL I. Moreover, previous studies on *M*. *leprae* adhesion to SC were always performed with killed bacteria that may in the end display altered cell-wall structures, and, therefore, lead to misleading conclusions regarding the relative importance of the different bacterial molecules involved in host-cell binding [12, 31, 41]. The use of a PGL I-expressing BCG strain made it possible to decipher the essential role played by PGL I in this process in view of the fact that the BCG WT strain expressing Hlp, but not PGL I, was unable to invade SC. In any case, it is deemed worthwhile to investigate the chance that, subsequent to the initial PGL-I-mediated *M*. *leprae-*SC interaction, the Hlp binding to laminin may provide the bacilli with higher avidity.

The present study likewise showed that CD206, an important mycobacterial recognition receptor [28, 32, 42–45], is significantly upregulated at both the mRNA and protein levels at early time points after SCs infection by PGL I-producing mycobacteria. When stimulus occurred with dead bacteria or PGL I-covered latex beads, this effect was not detected, suggesting that while PGL I binding to SC receptors is necessary, it is insufficient in terms of inducing this phenotypic change in SCs. This observation indicates that the status of bacilli (live or dead) is a key aspect in the interplay between *M*. *leprae* and SC and that live bacteria might modulate several pathways that go beyond the binding of PGL I to laminin 2 in these cells. Indeed, in previous studies, we have shown that only live *M*. *leprae* was able to induce the accumulation of lipids leading up to the formation of a foamy phenotype in infected SCs [23]. More recently, the capacity of *M*. *leprae* to modulate host-cell glucose metabolism and activate the IFN type I response in SCs has also been demonstrated to primarily depend on bacterial viability [21, 46].

CD206 or MR is a member of the C-type lectin family that binds mannose and fucose with the highest affinity [47]. MR is a pattern-recognition receptor (PRR) performing a key role in binding to microbial pathogens and facilitating their uptake by innate immune system cells. CD206 was shown to play a major role in the interaction of pathogenic mycobacteria with human macrophages, via recognition of Man-LAM abundantly present on their cell surface [45, 48–50]. Our findings suggest that the involvement of CD206 in the first wave of *M*. *leprae* entry in SCs is probably minimal, increasing in importance at later time points of infection. The observance of a lower *M*. *leprae*-invasion rate into SCs in which *mrc1* was silenced is indicative that the ManLAM-MR binding may participate in bacterial recognition and uptake after PGL I-mediated internalization. Moreover, infection of SCs for 48h in the presence of excess of free mannose reduced *M*. *leprae* internalization by about 50% (S9 Fig). Also, the induction of CD206 only by live *M*. *leprae* may at least partially explain the higher efficiency of live bacteria in comparison to dead organisms to enter into SCs.

Although we observed a clear upregulation of CD206 in infected SCs, the induction by *M*. *leprae* of additional receptors such as Dectin-2 or the dendritic cell-specific adhesion molecule 3-grabbing nonintegrin (DC-SIGN or CD209) cannot be ruled out since they also bind to ManLAM [51, 52]. Teles *et al*. [52] have furnished evidence that human SCs may express CD209, both *in vitro* and in neural leprosy lesions. Although *M*. *leprae* was also found in CD209^(-)^ SCs, it was shown to contribute to *M*. *leprae-*SC binding and be consistent with the detection of *M*. *leprae*-specific antigens *in vitro* and *in situ* in CD209^(+)^ SCs. The capacity of *M*. *leprae* to induce DC-SIGN expression is a topic deserving of further study.

We also showed the involvement of the nuclear receptor/transcriptional factor PPARγ in the MR induction by PGL I-producing mycobacteria [36, 53]. Moreover, a positive loop between CD206 and PPARγ was detected since blocking *M*. *leprae* recognition with excess of free mannose inhibited PPARγ activation. PPARγ is a master transcriptional factor regulating multiple cellular functions, including lipid metabolism and foam-cell generation [54–57]. The role of PPARγ and subsequent lipid-droplet biogenesis in mycobacterium-infected macrophages has also been described [37, 55]. Since *M*. *leprae* induces LD formation [23, 58], an obvious subsequent investigation was the involvement of PPARγ in LD formation. Of note, an important finding of the current study was that interference in the CD206/ PPARγ crosstalk, either by inhibiting bacterial recognition by mannose receptors or by blocking PPARγ activation completely abolished *M*. *leprae*- induced lipid droplets in SCs.

Next, we identified a dependence of PGE2 production on PPARγ activity in SCs. This observation, in combination with the known role of this nuclear receptor on LD formation [55], is in agreement with our previous studies indicating that PGE2 synthesis occurs in lipid droplets and its secretion is in correlation with the lipid droplet levels in *M*. *leprae* infected SCs [23]. Actually, in that same study we had identified a link between lipid metabolism through LD formation and the innate immune response triggered by live *M*. *leprae* in SCs. Besides PGE2, *M*. *leprae* was able to induce IL-10 that was abolished when LD formation was inhibited. Moreover, SCs even started producing IL-12 in the absence of LD formation. Interestingly, the same phenotype (decreased IL-10 and increased IL-12) was observed when NS-398, a COX-2 inhibitor, was used, suggesting that PGE2 may contribute to the negative modulation of the innate immune response toward intracellular infection. This goes in line with the immunomodulatory properties of PGE2, which has been shown to inhibit Th1 response and the microbicidal mechanisms of macrophages [59–62]. Furthermore, Schreiber *et al*. [63] found evidence that the mannose receptor biosynthesis is up-regulated by E-series prostaglandins. The hypothesis that a PGE2 autocrine loop may participate in raising MR expression levels in *M*. *leprae-*infected SC, needs more scrutiny.

In previous studies, we detailed the capacity of *M*. *leprae* to induce lipid accumulation in both macrophages and SCs [23, 58, 64]. In SCs, this effect was only observed with live bacteria, being in agreement with the findings here presented. Moreover, lipid droplets were shown to be recruited to mycobacterium-containing phagosomes, and both blockage of this recruitment or inhibition of LD formation lead to bacterial killing [23]. Moreover, as mentioned above, *M*. *leprae*-induced LD biogenesis and PGE2 production play a role in the generation of an innate immune response that may be permissive for bacterial persistence and proliferation. Thus, the decreased *M*. *leprae* viability in cells in which bacterial recognition through MR became inhibited after adding an excess of free mannose, can be explained by the blockage of LD formation and the secondary effects as a consequence of this inhibition.

In addition to aiding in bacterial internalization, signaling through MR/CD206 has also been seen to play an important role in *M*. *tuberculosis* pathogenesis [31, 50]. In infected macrophages, the recognition of ManLAM, abundantly present on the *M*. *tuberculosis* cell surface, by MR has been detected directing live bacilli to a phagosomal compartment with limited lysosomal fusion. This process was seen to be MR-ManLAM-specific because entry via another C-type lectin PRR, DC-SIGN, did not mediate this effect. Neither did PILAM, LM, nor any other mannosilated glycolipids present in the mycobacterial cell envelope [31]. In a recent study, the signaling cascade triggered by MR/CD206 to limit phagosome-lysosomal fusion was described [65]. In a previous report, we showed an active avoidance of phagolysosomal fusion in SCs by viable, but not heat-killed *M*. *leprae* [66]. Assuming that MR/CD206 triggers a similar signaling cascade in SCs, this could be explained by the fact that MR induction only occurs in the presence of live bacteria. However the link between *M*. *leprae* sensing via the CD206 and the limited phagosome maturation observed in *M*. *leprae* infected SC needs confirmation.

Our results imply that besides PGE2, PPARγ also regulates IL-8 production by SCs in response to *M*. *leprae*. Indeed, it has also been reported that the IL-8 promoter contains a PPARγ response element whose expression is regulated by PPARγ synthetic ligands [67]. Also, it was previously shown that lepromatous leprosy patients tend to have an up-regulated IL-8 response [68] and that SCs produce higher levels of IL-8 after contact with *M*. *leprae* antigens [69]. Moreover, our findings are in connection with a previous study that described that PPARγ induction by a virulent *M*. *tuberculosis* strain in human macrophages was seen to mediate the subsequent production of IL-8 and PGE2 by these cells [35]. IL-8 is a classic pro-inflammatory mediator that has been linked to demyelination and neurodegeneration [70]. Interestingly, *M*. *leprae*-induced demyelination as a strategy for intracellular survival has previously been described. The demyelination process induces de-differentiation of SCs and de-differentiated Schwann cells have been described as highly susceptible to *M*. *leprae* invasion [71]. IL-8 may also be taking part in cell recruitment and inflammation in these nerves.

MR expression may potentially influence multiple cellular functions due to the presence of an intronic miRNA, miR-511, embedded in and co-expressed with the *mrc1* gene in macrophages [42, 72]. miR-511-3p, the active strand of miR-511, is co-regulated with the MR mRNA and protein. Of note, it is predicted that miR-511-3p is in control of a wide range of genes involved in multiple cellular processes, including cellular morphogenesis, metabolism, protein localization, and gene transcription [73]. Interestingly, miRNA-511 presents a target sequence within the 3’ UTR of the human PPARγ gene [74]. It is recommended that further studies be carried out to explore the regulation and impact of miRNA-511 on *M*. *leprae-*infected SC and leprosy pathogenesis.

**Fig 11:**
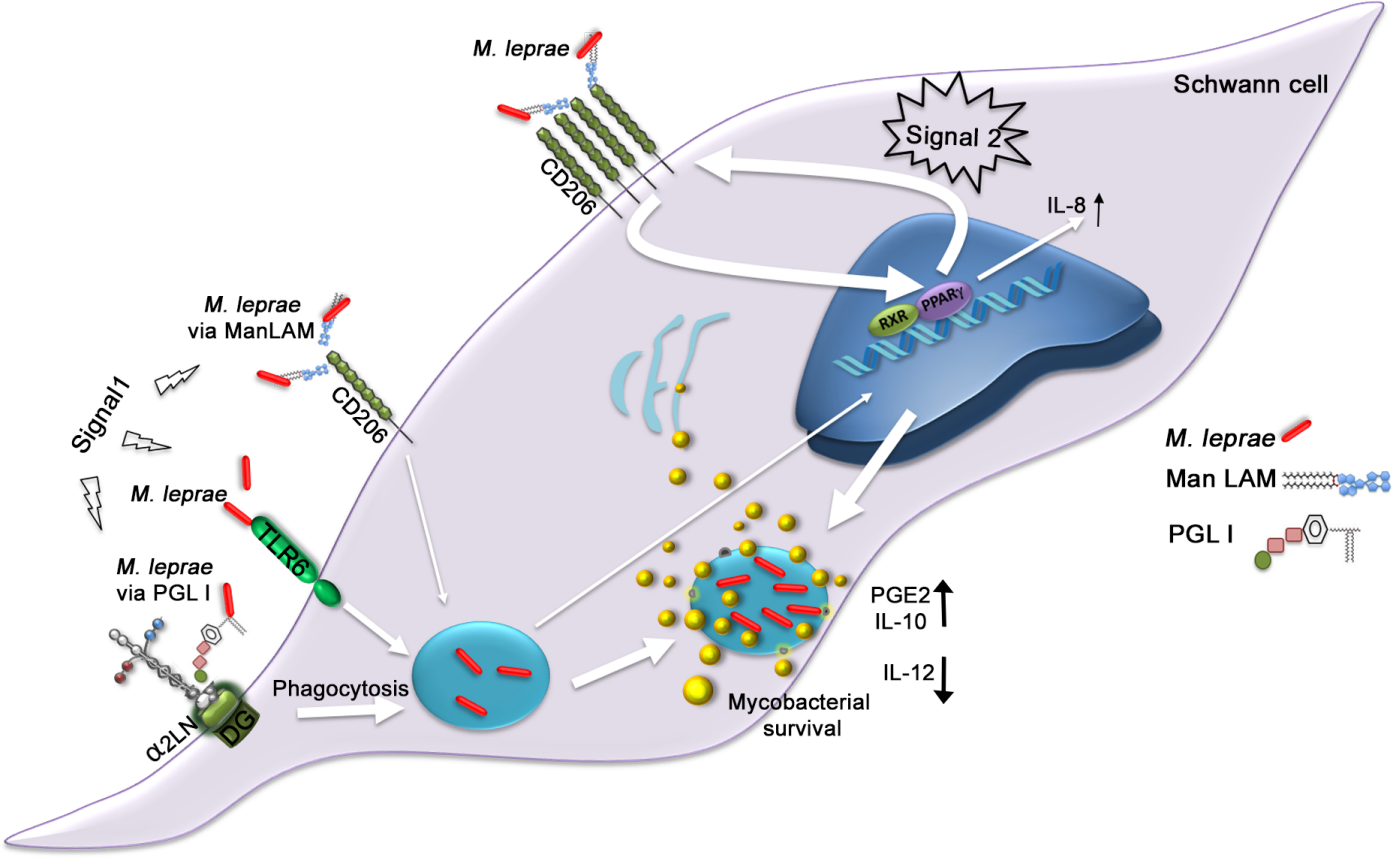
A model proposing a key role for the PGL I-induced CD206/PPARγ crosstalk in *M*. *leprae* neuropathogenesis. The first step (signal 1) of the pathway activation initiates through PGL I recognition via laminin-2 [12] allowing bacterial internalization. Bacterial sensing by TLR6 also contributes to bacterial entry [22]. Additionally, the recognition of *M*. *leprae* ManLAM by baseline levels of CD206 allows some bacterial entry and weak activation of PPARγ, with subsequent CD206 upregulation. The higher expression of CD206 results in increasing bacterial sensing by this pathway, triggering a stronger second signal (signal 2), where internalized *M*. *leprae* promotes the amplification of CD206/ PPARγ crosstalk, inducing the accumulation of LDs and their recruitment to bacterium-containing phagosomes. The higher levels of LDs promote the production of PGE-2 and IL-10 [22], which favors the inhibition of SC´s antimicrobial mechanisms. CD206/ PPARγ crosstalk also induces IL-8 secretion that may be involved in demyelination and neuroinflammation. Narrow arrows indicate the initials steps involved in *M*. *leprae*-SC interaction. Wide arrows indicate the major steps involved in the amplification loop of the PPAR/CD206 crosstalk. DG = Dystroglycan, α2 LN = alpha 2 laminin, RXR = Retinoid X Receptor.

In summary, the present along with the reported data in the literature strongly suggest that *M*. *leprae* actively modulates SC functions in order to establish a safe bacterial intracellular niche. A key fundamental aspect of *M*.*leprae* pathogenesis seems to be the induction of LDs. In the proposed model represented in Fig 11, signals from at least three recognition pathways contribute to LD formation: i) PGL-I, via binding to laminin-2, appears as the key molecule necessary for bacterial entry. Internalization was previously shown to be mandatory for LD formation [23]; ii) bacterial recognition via TLR6 was previously shown to play a role in *M*. *leprae* internalization and to be crucial for LD biogenesis, although the mycobacterial component that binds to TLR6 remains to be determined [22, 58]; and iii) signals generated from CD206 are also critical for LD induction. Binding of bacterial mannose enriched molecules to baseline levels of CD206 expressed by SCs is probably sufficient to initiate PPARγ activation, starting the reciprocal regulation with CD206 and their subsequent upregulation. The amplified signals originated from the CD206/ PPARγ crosstalk promotes the accumulation of host-derived lipids in infected cells. This is followed by LD recruitment to bacterium-containing phagosomes. The high levels of CD206 expression allows bacterial entry by this alternative pathway at later time points of infection. LD formation leads to PGE-2 and IL-10 secretion and inhibition of IL-12 production [22], which favors the inhibition of SC´s microbicidal mechanisms. Moreover, PPARγ activation induces the secretion of IL-8 that may contribute to demyelination as well as to the recruitment of immune cells to the site of infection and subsequent inflammation and tissue damage in infected nerves.

The data presented in this study disclose fundamental aspects of *M*. *leprae* pathogenesis in the nerve, pointing to the expression of PGL-I and the activation of the CD206/PPARγ crosstalk as central aspects in this process. The importance of CD206 in mycobacterial infection is further corroborated by evidence that CD206 polymorphisms are associated with an enhanced susceptibility to *M*. *tuberculosis* [75] or *M*. *leprae* infection [76]. Moreover, our results support the idea that SCs are immunocompetent cells that play active role during peripheral nerve injury. Finally, the disclosed data could prove useful in developing alternative interventions to prevent and treat leprosy neuropathy, based on the blockage of PGL-I- mediated bacterial internalization and/or inhibition of PPARγ activation and PG production.

## Materials and methods

### Human Schwann cells

The ST8814 tumor cell line generously donated by J. A. Flechter (Dana Farber Cancer Institute, Boston, MA, USA) originated from malignant schwannomas (neurofibromatosis type 1) of patients with neurofibromatosis type I. The cells were grown in RPMI 1640 medium (LGC, Biotecnologia) supplemented with 100 U/mL of penicillin, 100 μg/mL of streptomycin, 2 mM l-glutamine (LGC, Biotecnologia, SP, Brazil), and 10% fetal calf serum (FCS) (Cripion Biotecnologia LTDA) in a humidified 5% CO2 incubator at 37°C. They were then plated in complete RPMI medium in culture dishes (Nunc A/S, Roskide, Denmark) or in 24-well plates (Falcon, Franklin Lakes, NJ, USA) at a density of 40,000 cells per well and maintained in humidified air in an atmosphere of 5% CO2 at 37°C for 24 h. Prior to infection or stimulation, the cells received new RPMI medium supplemented with 2% FCS but no antibiotics. Assays with primary cultures of human Schwann cells (PHSC) were carried out with cells isolated from human spinal nerves that were purchased from ScienCell, Carlsbad, CA, USA (Cat.no. 1700). The PHSC were maintained in Schwann cell medium (SCM, Cat. no. 1701, ScienCell) supplemented with Schwann cell growth supplement (SCGS, Cat. no. 1752, ScienCell) according to the suppliers’ recommendations.

### Mycobacterial culture and growth medium

*M*. *leprae* Thai-53 purified from athymic BALB/c (*nu/nu)* mouse footpads was donated by the Lauro de Sousa Lima Institute, Bauru, São Paulo, Brazil. Recombinant *M*. *bovis* BCG strains with plasmids expressing PGL I, PGL TB, or the BCG Wild Type with an empty plasmid were kindly provided by Dr. Christophe Guilhot (Institut de Pharmacologie et Biologie Structurale, Toulouse, France). Also, GFP expressing recombinant strains were provided. Culture was processed as described by Tabouret *et al*. [15]. Briefly, *M*. *bovis* BCG Pasteur 1173P2 was cultured in Middlebrook 7H9 broth (DIFCO laboratories, USA) and supplemented with 10% ADC (0.2% dextrose, 0.5% bovine serum albumin fraction V, 0.0003% beef catalase) and 0.05% Tween 80 under constant agitation on a magnetic plate until the exponential phase or on solid Middlebrook 7H11 broth containing ADC and 0.005% oleic acid (OADC) (Becton Dickinson, Sparks, USA). For recombinant strains, kanamycin was added to the medium at the final concentration of 50μg/mL. For GFP expressing recombinant strains, kanamycin (Km) and hygromycin (Hyg) were added to the medium at the final concentration of 40μg/ml and 50μg/ml respectively. *M*. *smegmatis* (mc^2^ 155) was cultured in Middlebrook 7H9 broth (supplemented with 10% ADC and 0.05% Tween 80). For *in vitro* infections, all bacteria were resuspended in RPMI1640 medium before use. Part of each lot of mycobacteria received lethal gamma irradiation of 15 kilogray (Aceletron—Acelétrica Comércio e Representações Ltda., Rio de Janeiro, RJ, Brazil), as previously recommended [44]. Bacilli were counted according to Shepard and McRae [77] criteria; and bacillar viability was corroborated by way of LIVE/DEAD *Bac*Light Bacterial Viability Kits (Invitrogen, USA), according to the manufacturer’s instructions.

### Phagocytosis assays

Mycobacteria were labeled with a red (PKH26) or green (PKH67) fluorescent dye according to the manufacturer´s instructions (PKH26GL, PKH67GL, Sigma-Aldrich). For the phagocytosis assays, PKH-labeled or GFP expressing bacilli were added to the ST8814 cells in 24-well plates and maintained in a humidified 5% CO_2_ incubator at 33°C for 48 h in an antibiotic-free medium. SCs were infected with recombinant BCG strains, *M*. *smegmatis* or *M*. *leprae* at, unless otherwise stated, a multiplicity of infection of 50 (MOI 50:1). Phagocytosis was evaluated using flow cytometry and fluorescence microscopy. Cells were harvested with Trypsin 1x (LGC, Biotecnologia), washed, and fixed with 1% paraformaldehyde. For PKH67, green-labeled bacilli flow cytometry was carried out using the Accuri C6 (Accuri Cytometers, Inc.) FL1-A (green) channel and analyzed via *CFLow Plus*. To distinguish between adhered and internalized bacilli, the fluorescence of the externally-adhered ones was quenched with Trypan Blue (Sigma-Aldrich). For the microscopic assays, cells were plated (Corning, Fisher Scientific, USA) on cover slips in 24-well plates. Fluorescence microscopy used the Axio Observer Z1 (Carl Zeiss) microscope and AxioVision Rel. 4.8 software (Zeiss, Göttingen, Germany). The mean fluorescent signal value was quantified via open-source ImageJ software. (https://imagej.nih.gov/index.html; Research Services Branch, National Institute of Mental Health, National Institutes of Health, Bethesda, MD, USA). Protocol for PKH labeling available at: dx.doi.org/10.17504/protocols.io.phcdj2w. Protocol for phagocytosis assays available at: dx.doi.org/10.17504/protocols.io.pnpdmdn.

### Invasion assays with ManLAM or PGL I-coated beads

Green fluorescent polystyrene beads (Polysciences, Warrington, PA) (1μM diameter) were coated with 100 μg/mL ManLAM derived from *M*. *leprae* (NR-19348, BEI Resources) in sodium bicarbonate buffer (pH 9.6). After washing, the beads were blocked with 2% BSA in PBS. Alternatively, the beads were coated with 100 μg/mL PGL I derived from *M*. *leprae* (NR-19342, BEI Resources). The coating protocol was based on Ng, *et al*. [12] ManLAM or PGL I-coated beads were incubated with SC cultures for 48 h. The degree of association or internalization was determined by flow cytometry and visualized by fluorescence microscopy. Procedure for coating of beads with glycolipids available at: dx.doi.org/10.17504/protocols.io.pmrdk56.

### Co-infection or co-stimulation assays

The first stimulus, consisting of unlabeled bacteria or non-fluorescent latex beads, was always carried out with a low (10:1) multiplicity of infection (MOI) or proportion (beads:cell) one hour prior to the second stimulus. In these assays, live and dead bacteria were tested as pre-stimuli. When latex beads or dead bacilli were added, the term “proportion” was applied. The second stimulus consisted of PKH67- or PKH26-labeled bacteria or green fluorescent latex beads at a MOI or proportion of 50:1. All experiments were carried out during a 48 h period (unless otherwise stated) and analyzed using flow cytometry or fluorescence microscopy. In some cases, compounds like mannose at 100 μg/mL or 1000 μg/mL (112585 Sigma-Aldrich, USA) and GW9662 at 5 μM (CAS 22978-25-2, Cayman Chemical, USA) were added to the culture medium before the first stimulus. GW9962 is an irreversible antagonist of the transcription factor PPARγ. Optimal concentration of GW9662 to inhibit lipid body biogenesis was determined empirically for ST8814-SC. Based on previous reports [37] three concentrations (1 μM, 5μM and 10μM) of the drug were tested. Using fluorescence microscopy and Oil Red O labeling, we did not detect any effect on lipid body formation with 1 μM. The 5 μM concentration had a visible negative effect on lipid body formation. And with the 10 μM we readily observed a cytotoxic effect upon microscopic evaluation. To assess cell viability after using GW9662, cytotoxicity testing via MTT tetrazolium was performed. The 5 μM concentration showed no cytotoxic effect. Results are shown as S10 Fig.

### CD206 or PPARγ immunolabeling

CD206 or PPARγ expression was evaluated using flow cytometry and/or fluorescence microscopy. For the former, ST8814 cells were harvested using EDTA 5mM and then washed and fixed with 1% paraformaldehyde. Mannose receptor labeling was carried out with antibody anti-CD206 -FITC (clone15-2, Mouse IgG1, κ1, Biolegend). Negative controls were labeled with an FITC-conjugated isotype antibody. CD206 detection was performed 24 h post-infection using the Accuri C6 (Accuri Cytometers, Inc.) FL1-A channel. For fluorescence microscopy, the cover-slip cells were fixed and permeabilized with PBS containing paraformaldehyde 1% and saponin 0.1%. PPARγ detection was analyzed 48 h post-infection by way of the specific rabbit polyclonal antibody (H-100) SC-7196 (Santa Cruz Inc., USA) followed by incubation with goat IgG anti-rabbit conjugated with Alexa Fluor 555 A-21428 (Molecular Probes, USA) for immunofluorescence detection. Nuclei were evidenced by DAPI (4, 6-diamidino-2-phenylindole) staining (Sigma-Aldrich); and slides were mounted with Permafluor (Thermo Scientific, Waltham, MA) and analyzed via the AxiObserver Z1 Colibri microscope. Immunofluorescence was quantified using Open-Source ImageJ1 software (Research Services Branch, National Institutes of Health, Bethesda, MD, USA). Protocol for antigen labeling of eukaryotic cells and detection by flow cytometry available at: dx.doi.org/10.17504/protocols.io.pg8djzw. Protocol for antigen labeling of eukaryotic cultured cells and detection by fluorescence microscopy available at: dx.doi.org/10.17504/protocols.io.phadj2e.

### Transfection and gene-silencing experiments

For gene-expression knockdown, ST8814 cells were plated in complete RPMI medium at a density of 30,000 cells in a 24-well plate. Cells were transfected with a pre-designed Silencer Select siRNA for the *mrc1* gene (siRNA ID 106809 or siRNA ID 279717, # AM16708, Ambion Life Technologies, USA) or the control siRNA (# 4390874, Ambion). Lipofectamine 2000 (Life Technologies) was used as a vehicle in line with the manufacturer’s instructions. The cells were transfected with 20 pmol of siRNA in a 100μl transfection mix of Opti-MEM (Gibco) and lipofectamine and 400μl of complete RPMI medium under a protocol in compliance with the manufacturer’s instructions (Life Technologies). Twenty-four hours after transfection, the cells were infected with live *M*. *leprae* for 48 h in RPMI medium+ 2% FCS, followed by extraction of the nucleic acids and measurement of the mRNA by qRT-PCR.

### Western blot

Cellular lysates were obtained using a cold “RIPA” buffer (50mM Tris pH 7.5; 1% Nonidet p40; 0.25% sodium deoxycholate; 0.1% sodium dodecyl sulfate) containing protease inhibitors (Complete Inhibitor Cocktail Tablets Roche, USA). Proteins were quantified via BCA assay (Thermo Scientific, USA). A total of 20μg of protein/well were loaded onto a sodium dodecyl sulfate-polyacrylamide gel electrophoresis (SDS-PAGE) and transferred to nitrocellulose membranes (Bio-Rad, Hercules, CA, USA). The antibodies PPARγ (H-100) SC-7196 (rabbit) and GAPDH (H2114) SC-32233 (mouse), both from Santa Cruz Inc. (USA), were diluted in 5% (w/v) nonfat milk dissolved in Tris-Tween-buffered saline (TTBS; 20 mM Tris-HCl buffer, pH 7.6, containing 137 mM NaCl [v/v] 0.05% Tween 20). Results were visualized via the enhanced chemiluminescence detection system—ECL (Amersham Biosciences, NJ, USA). Protocol for Western Blot available at: dx.doi.org/10.17504/protocols.io.pnrdmd6.

### IL-8 and PGE2 measurements

After stimulation, culture supernatants were harvested, centrifuged, and stored at -70°C. IL-8 levels in the ST8814 culture supernatant were evaluated by ELISA using the DuoSet kit (R&D Systems). PGE2 concentration was measured in cell-free supernatants via an EIA kit (Cayman Chemical Co., Ann Arbor, MI, USA). The assays were conducted according to the manufacturer’s protocol.

### Lipid droplets staining and quantification

Schwann cells adhering to coverslips were fixed in 4% paraformaldehyde, and the area occupied by LDs were analyzed using fluorescent Oil red O (ORO, Cat. N° O0625 SIGMA). Coverslips were mounted, the morphology of the fixed cells was observed, and LDs areas were enumerated in 500 consecutively scanned cells. Nuclei were stained with 2 mM DAPI (Sigma-Aldrich) at room temperature for 5 min. The ORO images (taken with a 40-objective lens) were transformed into black and white pictures and analyzed via Open-Source ImageJ1 software. The spots were determined by automatic spot detection; and the total area and average size of fluorescent LDs was obtained for each field and divided by the number of cells in the respective field. Fluorescence microscopy used the Axio Observer Z1 (Carl Zeiss) microscope and AxioVision Rel. 4.8 software (Zeiss, Göttingen, Germany). Protocol for Oil Red O staining available at: dx.doi.org/10.17504/protocols.io.phbdj2n.

### Real-time quantitative reverse transcription PCR (qRT-PCR)

Total RNA was extracted from stimulated SCs using TRIzol (Thermo Fisher Scientific) according to the manufacturer’s instructions. Total RNA was converted to cDNA using the RevertAid first-strand cDNA synthesis kit (Thermo Fischer Scientific); and samples were stored at −20°C until further use. A total of 10ng of cDNA was used for qRT-PCR that was performed by StepOnePlus (Applied Biosystems, Foster City, CA, USA) and the SYBR Green PCR master mix (Applied Biosystems). Specific primers for *mrc1* were used (Forward: TGGTGGAAGAAGAAGCAGTC/ Reverse: TAGTCAAGGAAGGGTCGGAT). Thermal cycling conditions comprised an initial incubation at 95°C for 10 min, 40 cycles of denaturation at 95°C for 15 s, and annealing and extension at 60°C for 1 min. To normalize the relative *mrc1* expression, *rpl13* (Forward: GACAAGAAAAAGCGGATGGT /Reverse: GTACTTCCAGCCAACCTCGT) was used as an endogenous control whereas the relative expression values (*mrc1*/*rpl13*) were obtained by converting the cycle threshold (Ct) values according to the following formula: expression value = 2^(−ΔΔCt)^. Protocol for RNA extraction available at: dx.doi.org/10.17504/protocols.io.pg7djzn. Protocol for qPCR assays available at: dx.doi.org/10.17504/protocols.io.pnqdmdw.

### *M*. *leprae* viability

*M*. *leprae* viability was determined by qRT-PCR using the protocol previously described by Martinez, *et al* [78]. Briefly, ST8814 cells infected for 48 h with *M*. *leprae* and pre-incubated with mannose were submitted to RNA and DNA extraction with TRIzol (Thermo Fisher Scientific). DNA was removed from the RNA preparations using the DNA-free turbo kit (Ambion). RNA was reverse transcribed via a random primer and GoScript kit following the manufacturer’s instructions (Promega). The levels of 16S rRNA were determined and normalized against 16S DNA. PCR efficiency for each experiment was gauged via LinRegPCR software [79]; and normalization was performed considering efficiency corrections, as described above [80]. The same set of primers for the rRNA 16S was used for both cDNA and DNA: sense 5^’^- GCATGTCTTGTGGTGGAAAGC -3^’^ and antisense 5^’^- CACCCCACCAACAAGCTGAT -3^’^. cDNA and DNA were measured by TaqMan real-time PCR assay (Taqman probe 16S 5^’^- CATCCTGCACCGCA -3^’^). *M*. *leprae* viability was determined by the comparative Ct method [78]; and 100% viability was arbitrarily assumed for the infected control samples. Other values were normalized as a percentage of the control. Reactions were incubated in the ABI StepOne Plus Sequence Detection System (Applied Biosystems). Protocol for determining *M*. *leprae* molecular viability by qPCR available at: dx.doi.org/10.17504/protocols.io.pmwdk7e.

### Nerve biopsies and immunofluorescence

Histological sections of the peripheral nerves of five patients with pure neural leprosy (PNL) were included in the present study. One of them was a relapse of lepromatous leprosy. Diagnosis of PNL was carried out according to Jardim *et al*. [81] and all included cases presented acid-fast bacilli (AFB) in the biopsy. Also, three patients with axonal neuropathies not related to leprosy were included as controls. All patients were in attendance at the Leprosy Outpatient Unit of the Oswaldo Cruz Foundation, Rio de Janeiro, RJ, Brazil. After informed consent, nerve biopsy specimens were obtained and used for immunofluorescence testing. Tissue sections were thawed on sylane pre-coated slides and then rehydrated and stained with haematoxylin-eosin to evaluate the inflammatory infiltrate and cellularity. In accordance with standard protocols, the tissue sections were then stained with Gomori's trichrome to assess fibrosis and nerve structure [82] and subsequently submitted to Wade [39] staining to detect acid-fast bacilli. Immunofluorescence of cryostat tissue sections was performed initially, blocking the unspecific binding sites with 5% normal goat serum (NGS; Life technologies) with 2% bovine serum albumin (BSA; Sigma-Aldrich) in PBS containing 0.3% Triton X-100 (Amersham Biosciences) at room temperature for 4 h. After blocking, the tissue sections were incubated overnight with antibodies against S100 (rabbit polyclonal; Sigma-Aldrich) or anti-Myelin Basic Protein (anti-MBP Rat monoclonal; Millipore), two specific SC markers, lipoarabinomannan for *M*.*leprae* labeling (anti-LAM Rabbit polyclonal NR1809 provided by Bei Resources), and CD206 (clone19.2, Mouse IgG1,; BD Bioscience, San Jose, CA), a mannose receptor marker, followed by incubation for 2 h with goat anti-rabbit IgG conjugated with Alexa Fluor 568 A-11011, goat anti-mouse IgG1 conjugated with Alexa Fluor 488 A-21121 (Molecular Probes, Carlsbad, CA) and goat anti-rat IgG conjugated with Alexa Fluor 594 (Abcam, Cambridge, UK). As negative control the tissue sections were incubated only with secondary antibody, omitting the primary antibody. Nuclei were stained with DAPI (Sigma Aldrich). The immunofluorescence of peripheral nerve sections was examined using a Zeiss Axio Observer microscope (Carl Zeiss, Thornwood, NY, USA) equipped with Plan Apo 40 and 100 objectives and coupled with Apotome optical sectioning system to generate optical sections of the fluorescent images (Apotome.2- Carl Zeiss). The system was coupled to a CoolSNAP-Pro CF digital camera in conjunction with Axion Vision Version 4.7.2 software (Carl Zeiss). The images were edited via AxioVision software and their contrast was enhanced via Adobe Photoshop 14 (Adobe Systems). Protocol for immunohistochemistry for frozen sections: dx.doi.org/10.17504/protocols.io.pkmdku6.

### MTT assay

Assessment of the toxic effect of GW9662 on SCs was performed using an MTT assay. MTT, 3-(4,5-dimethylthiazol-2-yl)-2,5-diphenyl tetrazolium bromide (Sigma-Aldrich). The cells were seeded in 96-well plates at a density of 3000 cells per well and allowed to attach overnight. After a 44 h culture 10 μl MTT (5 mg/mL) was added to each well for 4 h. The reactions were terminated by removing all culture medium and adding 100 μL de SDS 10% (Bio-Rad) to each well. Following uniform oscillation for 10 min to ensure complete solubilization of the purple formazan crystals, the absorbance values were determined at 570 nm with a plate reader. Assessment of the toxic effect of SC infection with BCG or BCG PGL I at MOI 50:1 for 48h at 33°C was also performed. The results are shown in S10 Fig.

### Ethics statement

For the use of human samples, written informed consent was obtained from all patients and the procedures described were approved by the Ethics Committee of the Oswaldo Cruz Foundation (Approval number 546/10). These samples were obtained specifically for this study.

### Statistical analysis

Results are presented as the mean ± standard error of the mean (SEM) of independent experiments at a minimum in triplicate. Data were analyzed using the GraphPad Prism 5 Project (GraphPad Software, La Jolla, CA, USA). Unless otherwise stated, analyses were performed by applying one-way ANOVA and the Bonferroni post-test to compare the different groups. A p-value <0.05 was considered statistically significant.

## Supporting information

S1 Fig PGL I mediates mycobacterial adherence and internalization into Schwann cells. Bacterial association and internalization of PKH67 labeled BCG recombinant strains was determined by Flow Cytometry (FL1-A channel). **A.** ST8814 SC were treated with BCG WT, BCG PGL TB, BCG PGL I for 48 h at 33°C and MOI 50:1. The degree of internalization was determined after Trypan blue quenching of the fluorescence of externally adhered bacilli. The percentage of cells with associated bacilli (adhered + internalized) was plotted in green and the percentage with internalized bacilli in blue. **B.** The degree of bacterial adherence at low temperature and short incubation time was determined by flow cytometry. ST8814 SC were treated with BCG WT, BCG PGL TB, BCG PGL I for 4 h at 4°C and MOI 50:1. The percentage of cells with adhered bacilli was plotted in green. **C.** ST8814 SC were treated with GFP expressing BCG WT, BCG PGL TB or BCG PGL I for 48 h at 33°C and MOI 50:1. The degree of association was determined by fluorescence microscopy. The percentage of cells with associated bacilli was plotted. Each result represents the mean ± SEM from three independent experiments. An ANOVA test followed by Bonferroni as a post test was performed and used for statistical analysis. ***p<0.001.
(TIF)

S2 Fig Live BCG PGL I or *M*. *leprae* are more efficiently internalized by Schwann cells compared to dead bacilli. **A.** Internalization degree of live and dead recombinant BCG strains was determined by flow cytometry after 48 h of incubation at 33°C and MOI 50:1. Bacteria were labeled with PKH67 and the degree of internalization was determined after Trypan Blue quenching. Results were represented as MFI. **B and C.** Internalization of live and dead *M*. *leprae* was determined by flow cytometry after 48 h of incubation at 33°C and different MOIs. ST8814 SCs were either left untreated (NI) or treated with PKH67 labeled bacteria and the degree of internalization was determined after Trypan Blue quenching. A representative histogram plot of the 48 h incubation experiment is shown. Results were represented as percentage of cell population with internalized bacteria or MFI of the cell population. Each result represents the mean ± SEM from three independent experiments. An ANOVA test followed by Bonferroni as a post test was performed and used for statistical analysis. *p < 0.05; ***p<0.001.
(TIF)

S3 Fig The expanded phagocytic capacity induced by *M*. *leprae* and BCG PGL I on SC is specific to BCG WT and is not induced by PGL I alone. **A.** Flow cytometry result showing no change in the degree of internalization of PKH67 labeled *M*. *bovis* BCG or latex beads when adding pure PGL I (15ng or 30ng) to the culture medium. **B.** Flow cytometry result showing no change in the degree of internalization of green fluorescent beads after a pre-stimulus with BCG PGL I or *M*. *leprae* at MOI 10:1.
(TIF)

S4 Fig Competition assay suggesting the mannose receptor (CD206) as a receptor candidate to mediate the internalization of BCG WT in Schwann cells. Representative images of fluorescence microscopy showing PKH 67 labeled-BCG WT association to SC after pre-infection with *M*. *leprae* and in presence or absence of mannose. Cells on coverslips were fixed with paraformaldehyde and stained with DAPI (blue) for nuclear localization. The addition of mannose at 1000 μg/mL to the culture medium reduced the BCG WT association rate 48 h post-infection. Results represent three independent biological replicates. Scale (white line) represents 10 μm.
(TIF)

S5 Fig Effect of GW9662 on *M*. *leprae* and BCG PGL I internalization into Schwann cells. Flow cytometry results showing the degree of bacterial internalization of live PKH67 labeled bacilli after 24 h (A) and 48 h (B) of incubation with SC at 33°C, MOI 50:1 in the presence or absence of GW9662 (5 μM). A. A representative histogram plot of the 24 h incubation assay shows fluorescence at the FL1-A channel. The addition of GW9962 (5 μM) to the culture medium had no significant effect on the internalization rate of BCG PGL I or *M*. *leprae* after 24h of incubation. B. The addition of GW9962 (5 μM) to the culture medium reduced BCG PGL I or *M*. *leprae* internalization rate 48 h post-infection. Each bar represents the mean ± SEM from at least three independent experiments in triplicate. An ANOVA test followed by Bonferroni as a post-test were performed and used for statistical analyses. *p < 0.05.
(TIF)

S6 Fig Effect of *mrc1* silencing on PGE2 production in infected and non infected Schwann cells. SCs were transfected for 24 h with control siRNA or siRNA targeting *mrc1*, followed by infection with *M*. *leprae*, BCG PGL I or BCG WT for 48 h. Supernatants were analyzed for PGE2 production by EIA. Unexpectedly, silencing of *mrc1* was shown to increase PGE2 production in the non infected condition.
(TIF)

S7 Fig CD206 is not up-regulated nor colocalizes with Schwann cells in control nerve lesions. Serial sections of nerve biopsies from patients (n = 2) with non-leprosy peripheral neuropathies were analyzed. **A.** Peripheral nerve tissue was labeled with antibodies for the SC-specific marker S100 (red image), and the mannose receptor CD206 (green image) and then visualized by fluorescence microscopy. The merged images show no CD206/S100 colocalization. Nuclei were labeled with DAPI (blue image). **B.** The corresponding isotype controls for CD206 and S100 were used as negative control. Scale bar 20μm. Insets: magnified views of CD206/S100 staining. Scale bar, 10μm.
(TIF)

S8 Fig CD206 colocalizes with Schwann cells in nerve lesions of leprosy patients. Serial sections of leprosy patients (n = 4) nerve biopsies were analyzed. Peripheral nerve tissue was labeled with antibodies for the SC-specific marker S100 (red image), and the mannose receptor CD206 (green image) and then visualized by fluorescence microscopy. Insets are magnified to view CD206/S100 co-staining SC. Nuclei were labeled with DAPI (blue image). Scale bar 10μm.
(TIF)

S9 Fig Competition assay suggesting the mannose receptor (CD206) as a receptor candidate to mediate the mycobacterial internalization into Schwann cells. The addition of mannose at 100 or 1000 μg/mL in the culture medium reduced *M*. *leprae* or BCG PGL I internalization rate 48 h post-infection at 33°C and MOI 50:1. MFI was determined using the flow cytometry FL1-A channel. In all experiments, the degree of internalization of PKH67-labeled bacilli was determined after Trypan Blue quenching. Results are represented as mean ± SEM of at least three independent biological replicates; and statistical significance was calculated by ANOVA followed by Bonferroni’s multiple comparison test. *p < 0.05; ** p<0.01.
(TIF)

S10 Fig Evaluation of GW9962 and BCG cytotoxicity on Schwann cells *in vitro* using MTT assay. **A.** SCs were incubated with GW9662 at 5 μM and 10 μM for 48 h and cell viability was determined by MTT assay. **B.** ST8814 SC were either left uninfected (NI) or were treated with BCG WT or BCG PGL I. After 48 h of incubation at 33°C and MOI 50:1, cell viability was determined by MTT assay. Each result is shown as mean ± SD of three assays. MTT, 3-(4,5-dimethylthiazol-2-yl)-2,5-diphenyl tetrazolium bromide (Sigma-Aldrich).
(TIF)
