# Supplementary figures and images for: PGL I expression in live bacteria allows activation of a CD206/PPARγ cross-talk that may contribute to successful Mycobacterium leprae colonization of peripheral nerves
Source: PLoS Pathog. 2018 Jul 6;14(7):e1007151. doi: 10.1371/journal.ppat.1007151 (PMC6056075; doi:10.1371/journal.ppat.1007151)

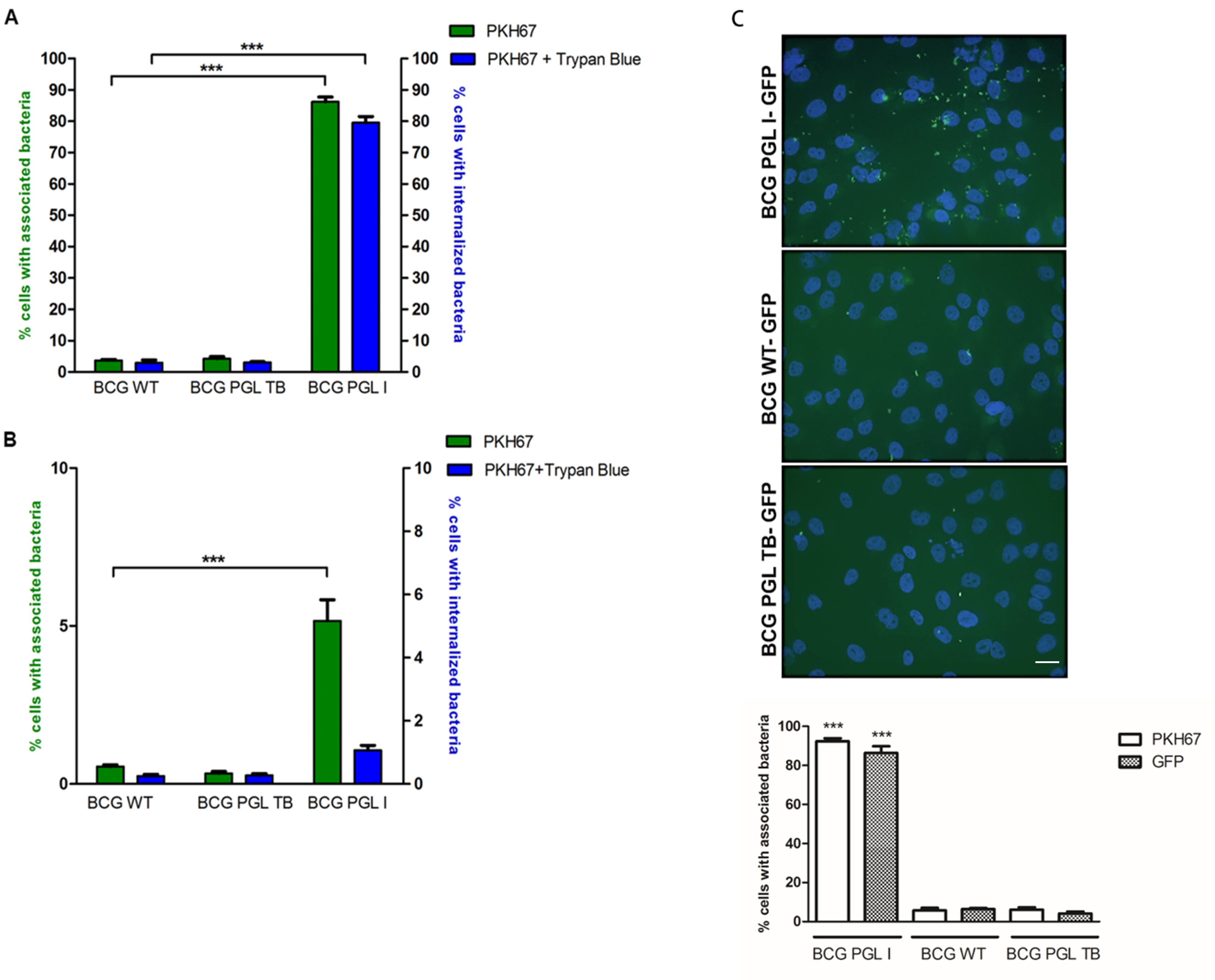

Supplement: S1 Fig — Bacterial association and internalization of PKH67 labeled BCG recombinant strains was determined by Flow Cytometry (FL1-A channel). A. ST8814 SC were treated with BCG WT, BCG PGL TB, BCG PGL I for 48 h at 33°C and MOI 50:1. The degree of internalization was determined after Trypan blue quenching of the fluorescence of externally adhered bacilli. The percentage of cells with associated bacilli (adhered + internalized) was plotted in green and the percentage with internalized bacilli in blue. B. The degree of bacterial adherence at low temperature and short incubation time was determined by flow cytometry. ST8814 SC were treated with BCG WT, BCG PGL TB, BCG PGL I for 4 h at 4°C and MOI 50:1. The percentage of cells with adhered bacilli was plotted in green. C. ST8814 SC were treated with GFP expressing BCG WT, BCG PGL TB or BCG PGL I for 48 h at 33°C and MOI 50:1. The degree of association was determined by fluorescence microscopy. The percentage of cells with associated bacilli was plotted. Each result represents the mean ± SEM from three independent experiments. An ANOVA test followed by Bonferroni as a post test was performed and used for statistical analysis. ***p<0.001. (TIF) [file ppat.1007151.s001.tif]

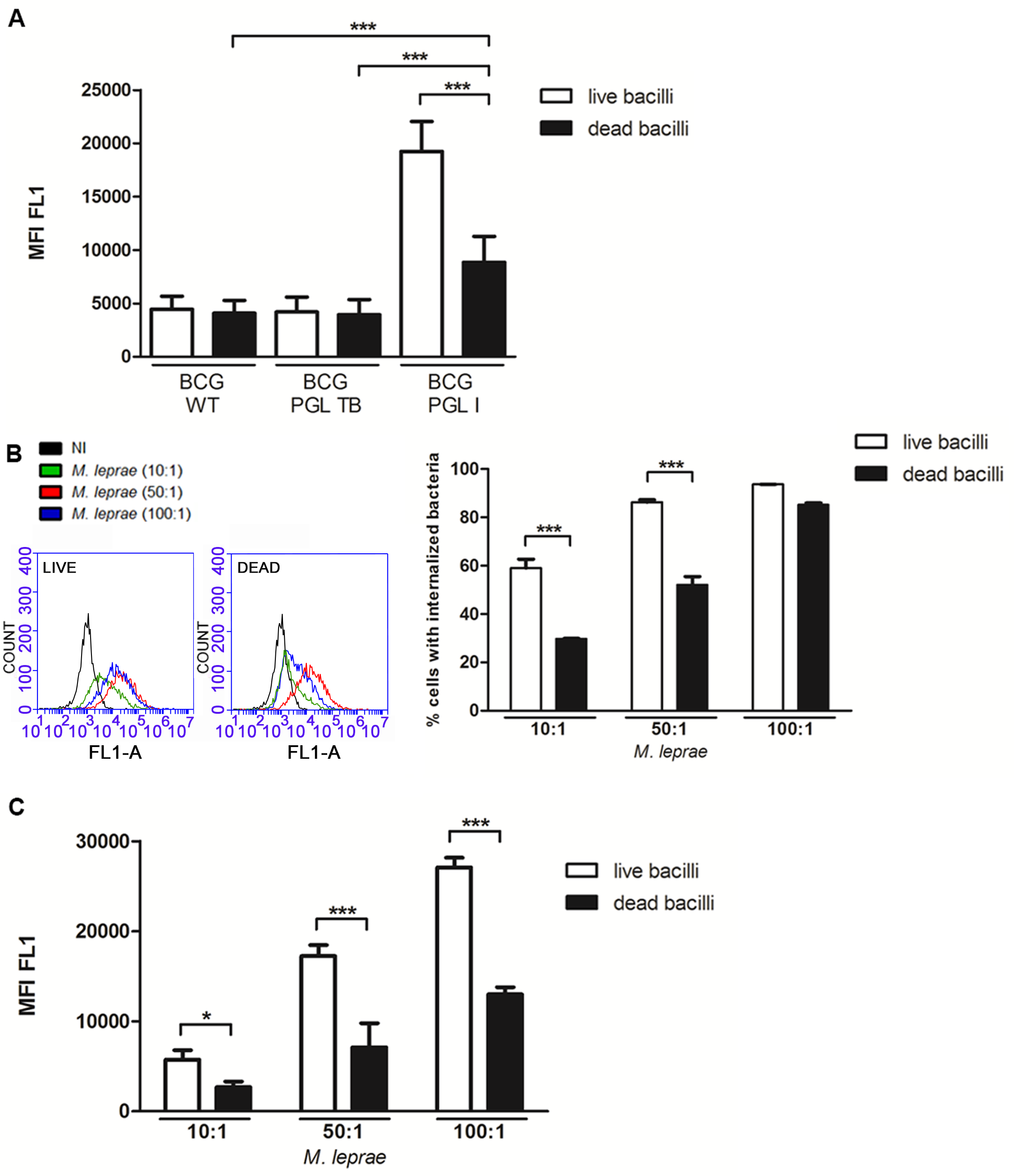

Supplement: S2 Fig — A. Internalization degree of live and dead recombinant BCG strains was determined by flow cytometry after 48 h of incubation at 33°C and MOI 50:1. Bacteria were labeled with PKH67 and the degree of internalization was determined after Trypan Blue quenching. Results were represented as MFI. B and C. Internalization of live and dead M. leprae was determined by flow cytometry after 48 h of incubation at 33°C and different MOIs. ST8814 SCs were either left untreated (NI) or treated with PKH67 labeled bacteria and the degree of internalization was determined after Trypan Blue quenching. A representative histogram plot of the 48 h incubation experiment is shown. Results were represented as percentage of cell population with internalized bacteria or MFI of the cell population. Each result represents the mean ± SEM from three independent experiments. An ANOVA test followed by Bonferroni as a post test was performed and used for statistical analysis. *p < 0.05; ***p<0.001. (TIF) [file ppat.1007151.s002.tif]

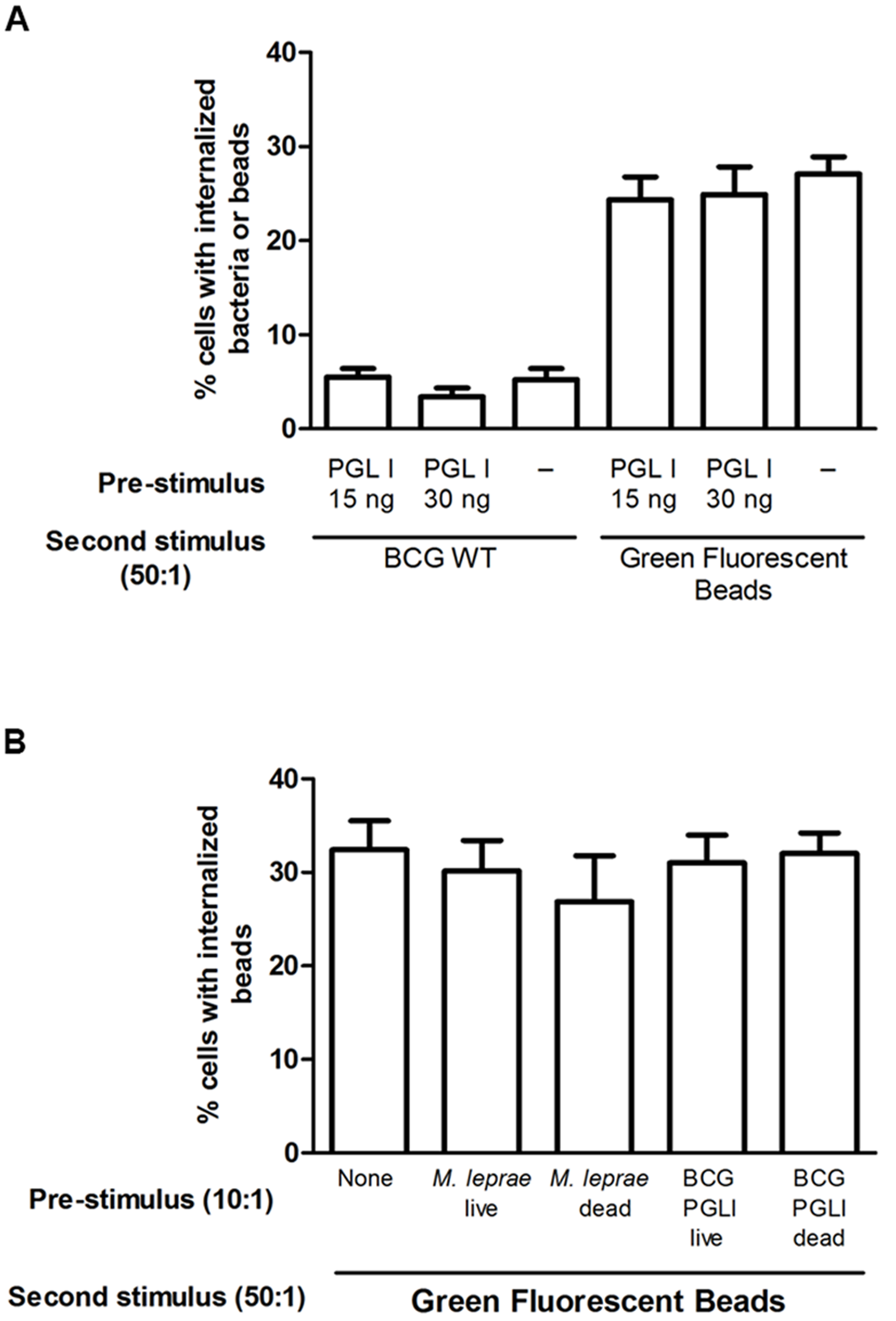

Supplement: S3 Fig — A. Flow cytometry result showing no change in the degree of internalization of PKH67 labeled M. bovis BCG or latex beads when adding pure PGL I (15ng or 30ng) to the culture medium. B. Flow cytometry result showing no change in the degree of internalization of green fluorescent beads after a pre-stimulus with BCG PGL I or M. leprae at MOI 10:1. (TIF) [file ppat.1007151.s003.tif]

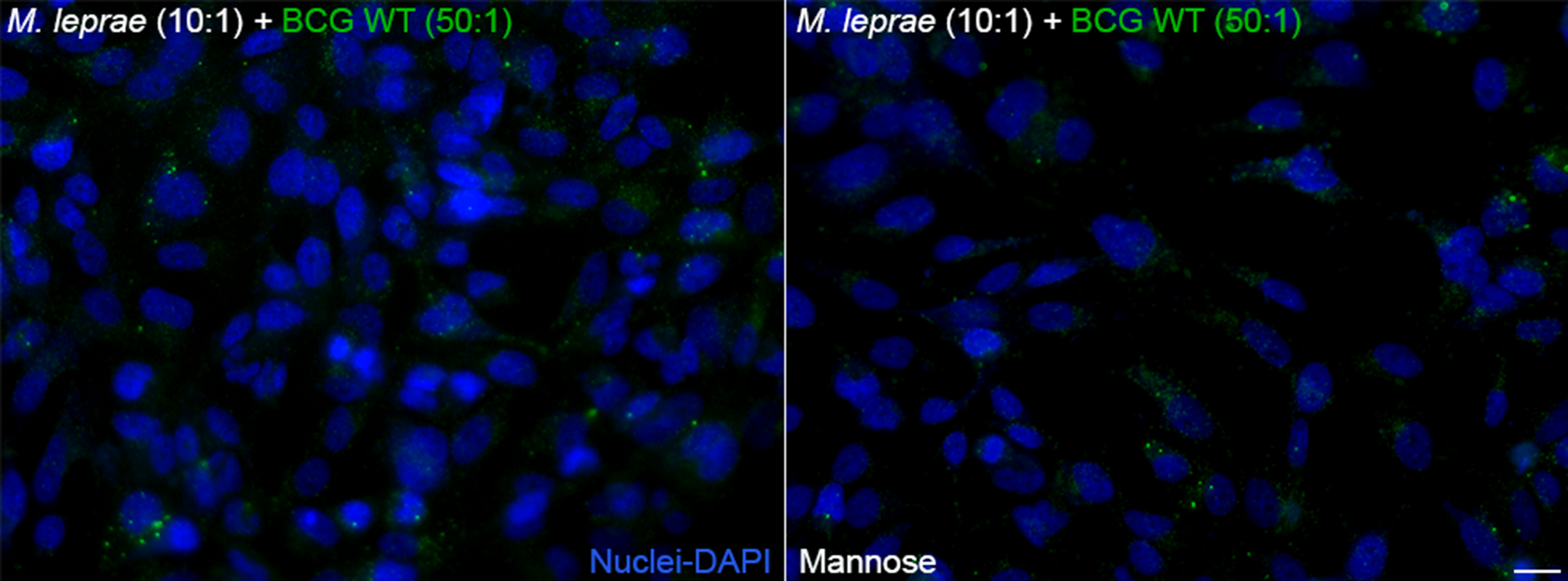

Supplement: S4 Fig — Representative images of fluorescence microscopy showing PKH 67 labeled-BCG WT association to SC after pre-infection with M. leprae and in presence or absence of mannose. Cells on coverslips were fixed with paraformaldehyde and stained with DAPI (blue) for nuclear localization. The addition of mannose at 1000 μg/mL to the culture medium reduced the BCG WT association rate 48 h post-infection. Results represent three independent biological replicates. Scale (white line) represents 10 μm. (TIF) [file ppat.1007151.s004.tif]

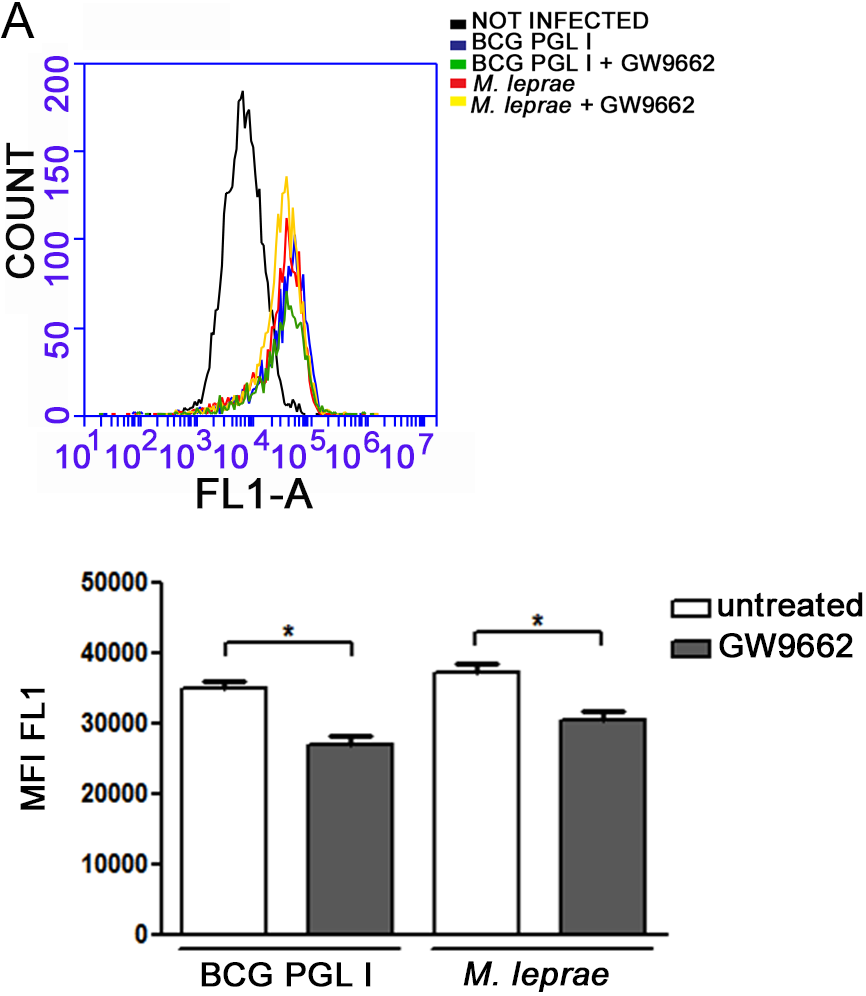

Supplement: S5 Fig — Flow cytometry results showing the degree of bacterial internalization of live PKH67 labeled bacilli after 24 h (A) and 48 h (B) of incubation with SC at 33°C, MOI 50:1 in the presence or absence of GW9662 (5 μM). A. A representative histogram plot of the 24 h incubation assay shows fluorescence at the FL1-A channel. The addition of GW9962 (5 μM) to the culture medium had no significant effect on the internalization rate of BCG PGL I or M. leprae after 24h of incubation. B. The addition of GW9962 (5 μM) to the culture medium reduced BCG PGL I or M. leprae internalization rate 48 h post-infection. Each bar represents the mean ± SEM from at least three independent experiments in triplicate. An ANOVA test followed by Bonferroni as a post-test were performed and used for statistical analyses. *p < 0.05. (TIF) [file ppat.1007151.s005.tif]

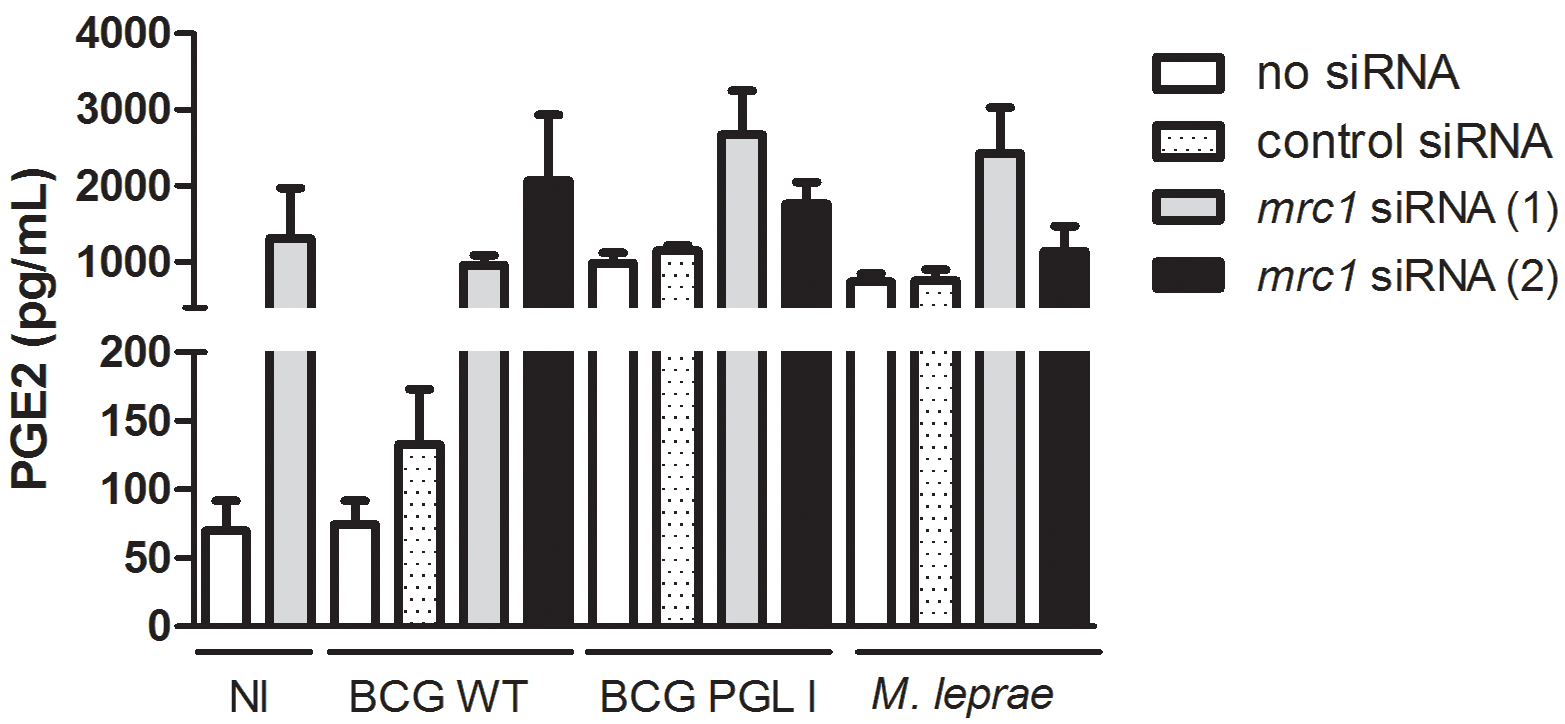

Supplement: S6 Fig — SCs were transfected for 24 h with control siRNA or siRNA targeting mrc1, followed by infection with M. leprae, BCG PGL I or BCG WT for 48 h. Supernatants were analyzed for PGE2 production by EIA. Unexpectedly, silencing of mrc1 was shown to increase PGE2 production in the non infected condition. (TIF) [file ppat.1007151.s006.tif]

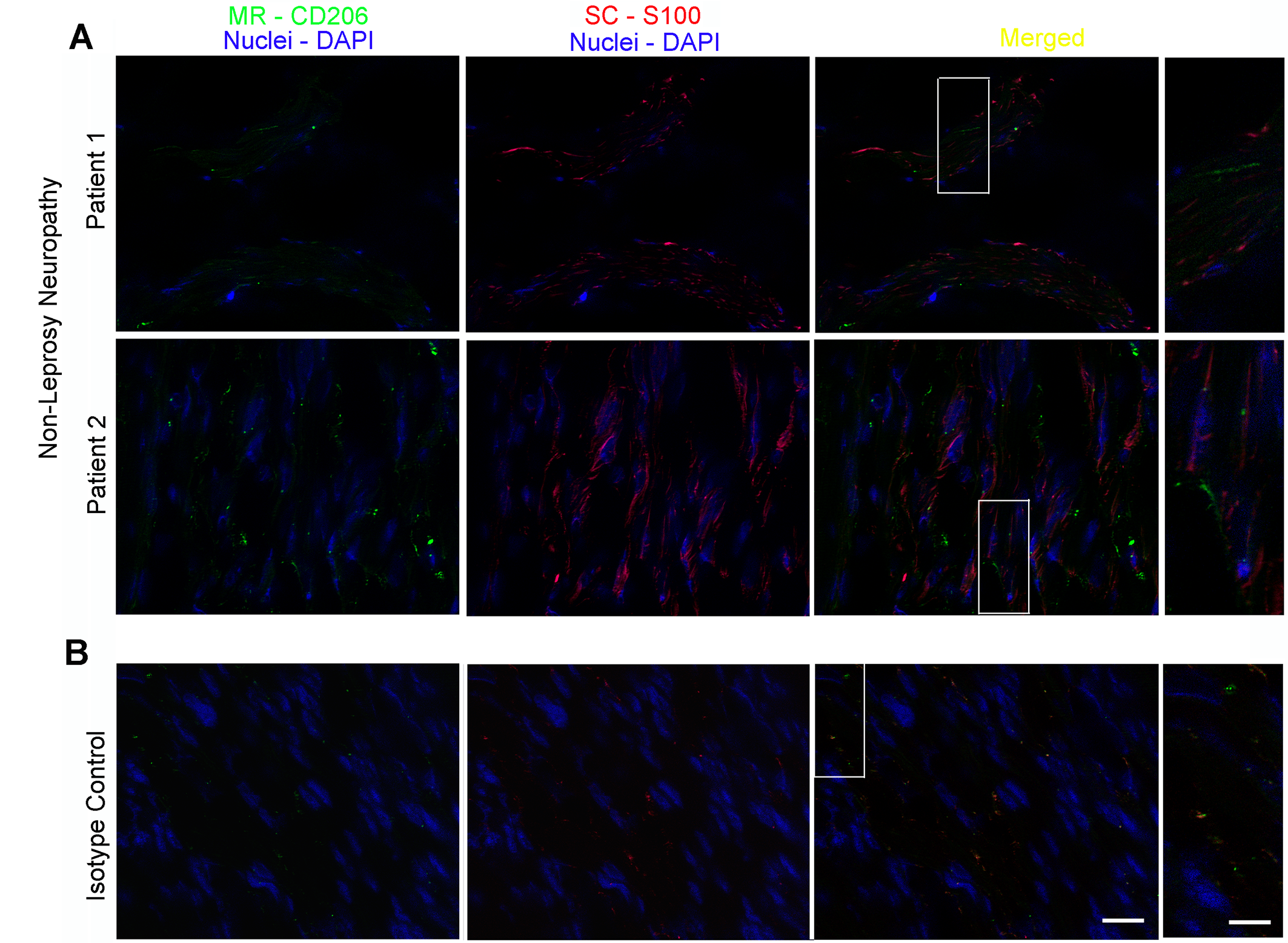

Supplement: S7 Fig — Serial sections of nerve biopsies from patients (n = 2) with non-leprosy peripheral neuropathies were analyzed. A. Peripheral nerve tissue was labeled with antibodies for the SC-specific marker S100 (red image), and the mannose receptor CD206 (green image) and then visualized by fluorescence microscopy. The merged images show no CD206/S100 colocalization. Nuclei were labeled with DAPI (blue image). B. The corresponding isotype controls for CD206 and S100 were used as negative control. Scale bar 20μm. Insets: magnified views of CD206/S100 staining. Scale bar, 10μm. (TIF) [file ppat.1007151.s007.tif]

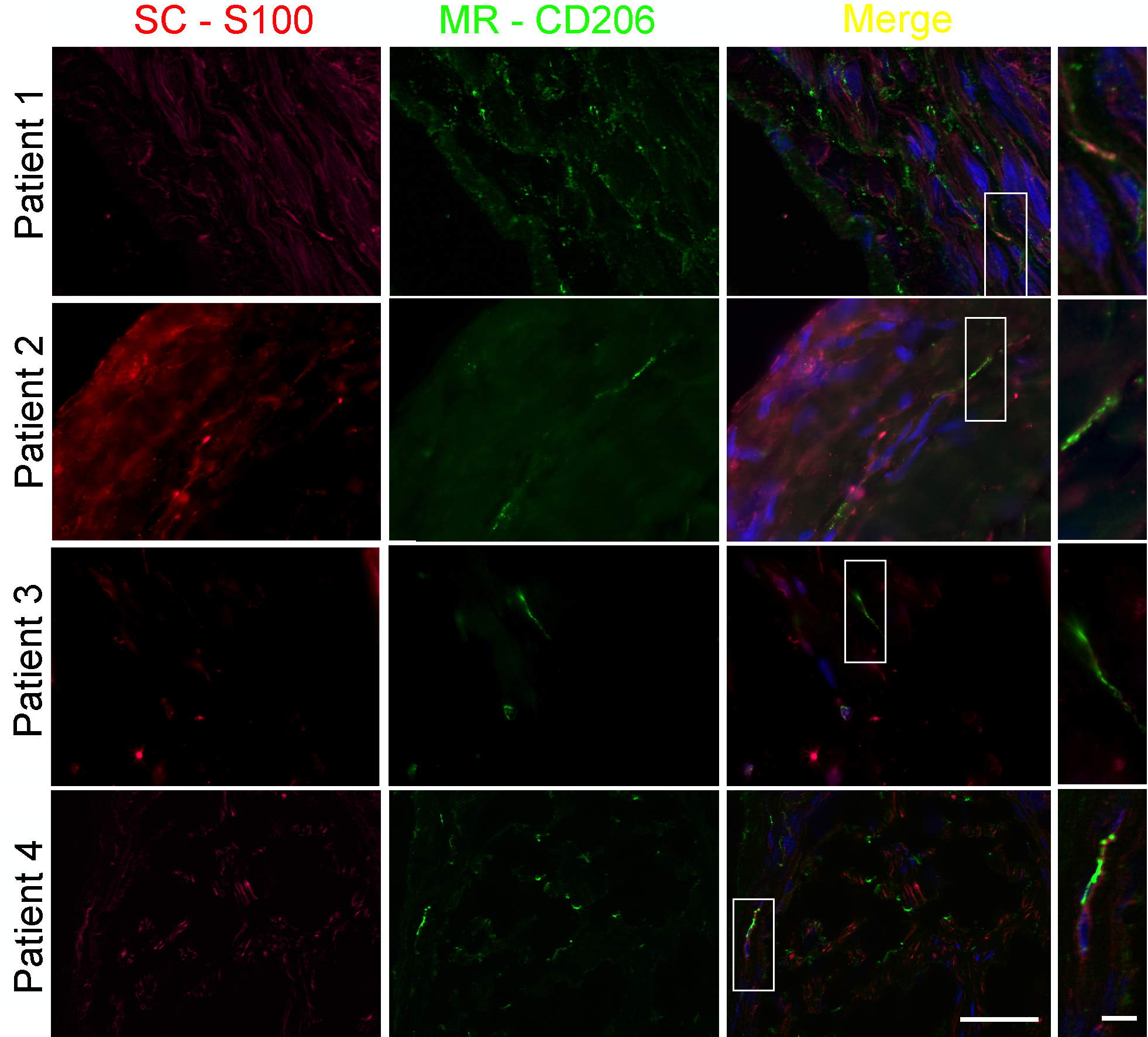

Supplement: S8 Fig — Serial sections of leprosy patients (n = 4) nerve biopsies were analyzed. Peripheral nerve tissue was labeled with antibodies for the SC-specific marker S100 (red image), and the mannose receptor CD206 (green image) and then visualized by fluorescence microscopy. Insets are magnified to view CD206/S100 co-staining SC. Nuclei were labeled with DAPI (blue image). Scale bar 10μm. (TIF) [file ppat.1007151.s008.tif]

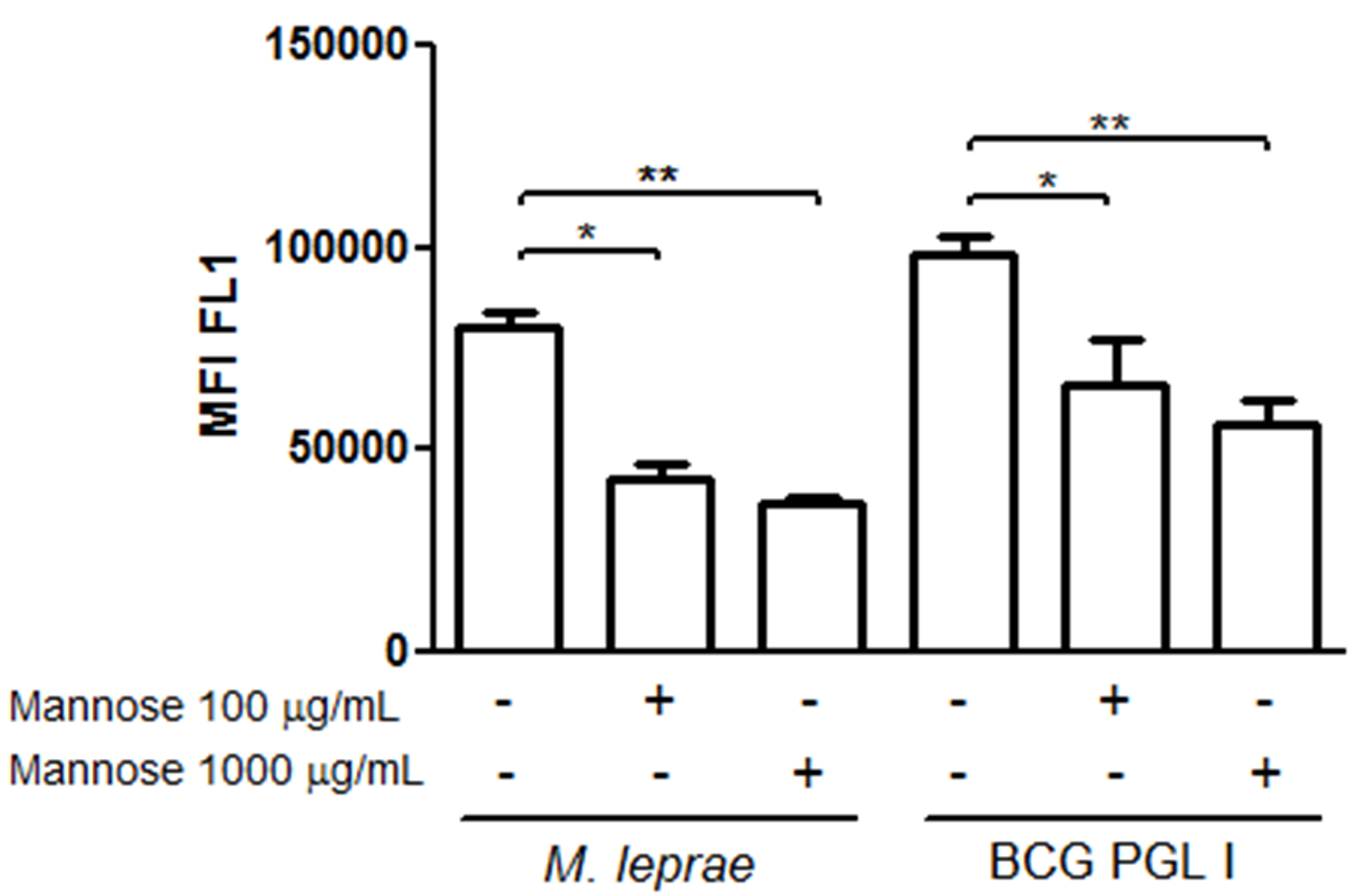

Supplement: S9 Fig — The addition of mannose at 100 or 1000 μg/mL in the culture medium reduced M. leprae or BCG PGL I internalization rate 48 h post-infection at 33°C and MOI 50:1. MFI was determined using the flow cytometry FL1-A channel. In all experiments, the degree of internalization of PKH67-labeled bacilli was determined after Trypan Blue quenching. Results are represented as mean ± SEM of at least three independent biological replicates; and statistical significance was calculated by ANOVA followed by Bonferroni’s multiple comparison test. *p < 0.05; ** p<0.01. (TIF) [file ppat.1007151.s009.tif]

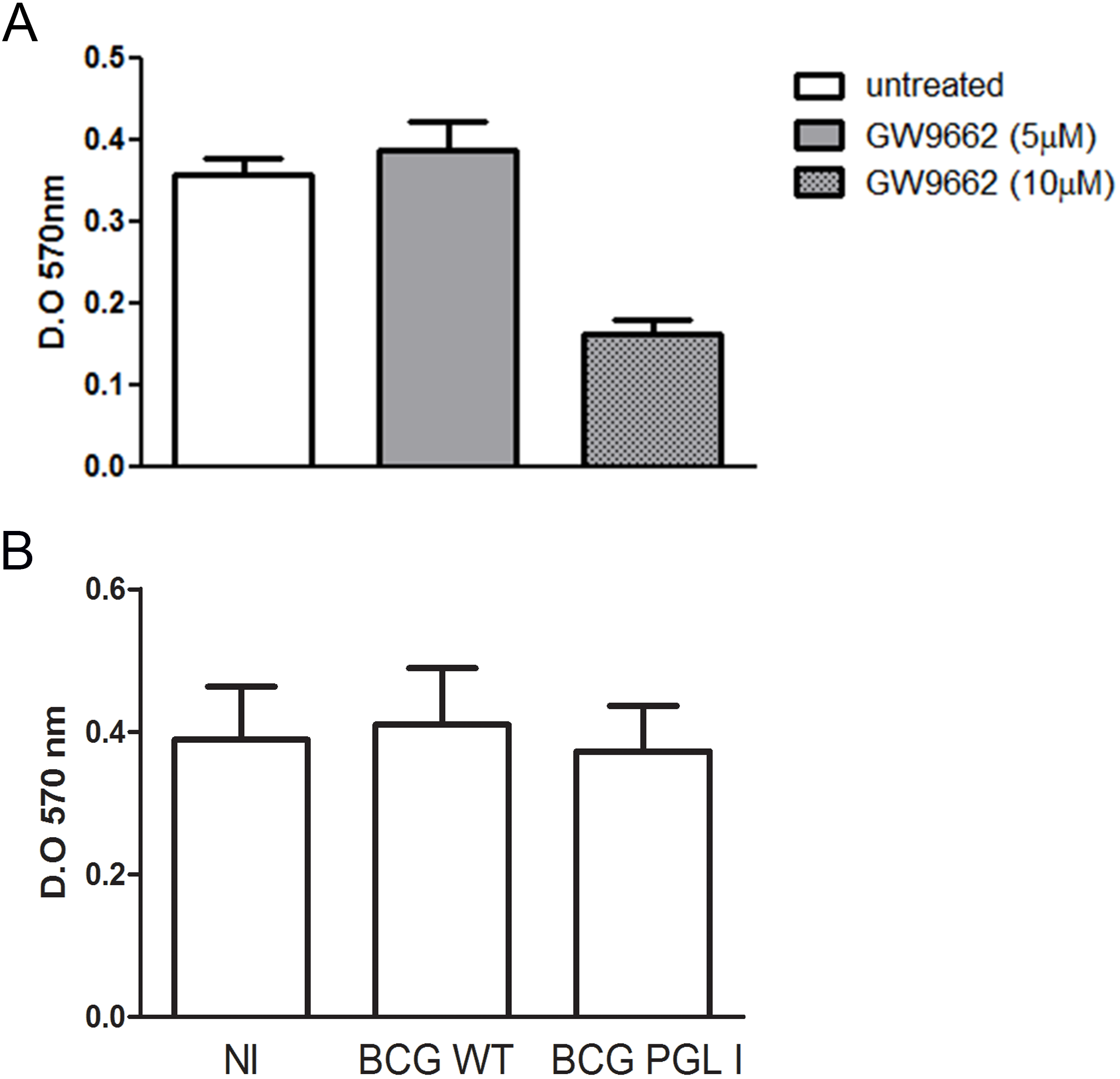

Supplement: S10 Fig — A. SCs were incubated with GW9662 at 5 μM and 10 μM for 48 h and cell viability was determined by MTT assay. B. ST8814 SC were either left uninfected (NI) or were treated with BCG WT or BCG PGL I. After 48 h of incubation at 33°C and MOI 50:1, cell viability was determined by MTT assay. Each result is shown as mean ± SD of three assays. MTT, 3-(4,5-dimethylthiazol-2-yl)-2,5-diphenyl tetrazolium bromide (Sigma-Aldrich). (TIF) [file ppat.1007151.s010.tif]
